# Exposure to urban and rural contexts shapes smartphone usage behavior

**DOI:** 10.1093/pnasnexus/pgad357

**Published:** 2023-11-28

**Authors:** Anna Sapienza, Marita Lítlá, Sune Lehmann, Laura Alessandretti

**Affiliations:** Department of Applied Mathematics and Computer Science, Technical University of Denmark, Kongens Lyngby 2800, Denmark; Copenhagen Center for Social Data Science, University of Copenhagen, Copenhagen K 1353, Denmark; Department of Applied Mathematics and Computer Science, Technical University of Denmark, Kongens Lyngby 2800, Denmark; Department of Applied Mathematics and Computer Science, Technical University of Denmark, Kongens Lyngby 2800, Denmark; Copenhagen Center for Social Data Science, University of Copenhagen, Copenhagen K 1353, Denmark; Department of Applied Mathematics and Computer Science, Technical University of Denmark, Kongens Lyngby 2800, Denmark

**Keywords:** smartphone data, digital behavior, urban–rural divide, quasi-experimental design

## Abstract

Smartphones have profoundly changed human life. Nevertheless, the factors that shape how we use our smartphones remain unclear, in part due to limited availability of usage-data. Here, we investigate the impact of a key environmental factor: users’ exposure to urban and rural contexts. Our analysis is based on a global dataset describing mobile app usage and location for ∼500,000 individuals. We uncover strong and nontrivial patterns. First, we confirm that rural users tend to spend less time on their phone than their urban counterparts. We find, however, that individuals in rural areas tend to use their smartphones for activities such as gaming and social media. In cities, individuals preferentially use their phone for activities such as navigation and business. Are these effects (1) driven by differences between individuals who choose to live in urban vs. rural environments or do they (2) emerge because the environment itself affects online behavior? Using a quasi-experimental design based on individuals that move from the city to the countryside—or vice versa—we confirm hypothesis (2) and find that smartphone use changes according to users’s environment. This work presents a quantitative step forward towards understanding how the interplay between environment and smartphones impacts human lives. As such, our findings could provide information to better regulate persuasive technologies embedded in smartphone apps. Further, our work opens the door to understanding new mechanisms leading to urban/rural divides in political and socioeconomic attitudes.

**Significance Statement**

While smartphones have become an essential part of people’s day-to-day life, little is known about which factors impact their usage. Using a dataset of 500K anonymized users from a range of countries, we investigate how exposure to urban/rural environments shape smartphone use. Since the urban/rural divide tightly correlates with socioeconomic and political boundaries, this is an important split. We find that, although individuals living in the countryside use smartphones less than their urban counterparts, they tend to use them for activities related to gaming and social media. Using a quasi-experimental design, we further show that these differences emerge because the place where people live directly affects smartphone use. Our findings can help design regulations and raise better awareness about smartphone use.

## Introduction

Smartphones have dramatically changed the reality we experience [1, 2]. More than a third of smartphone owners report that the phone is the first thing they reach for when they wake up [3, 4], and almost 50% report that they use their phones during the night [4] Increasing evidence shows that using smartphones impacts many aspects of our lives—from how we sleep [5] and exercise [6], to how we learn [7] and interact [8]. Some studies have highlighted that smartphone usage can lead to negative consequences, including damaging cognitive abilities [9], social interactions [10], mental [11, 12] and physical [13, 14] health. Other studies have reported the beneficial effects of smartphones [15] that contribute to building a sense of belonging [16], reduce social isolation [17], and improve psychological health [18]. These contradictory findings have been explained in light of how smartphones are used. Using the phone as a vehicle for entertainment and replacement for in-person companionship via online social networks is positively associated with problematic smartphone usage [19, 20] and low sense of meaningfulness [21], while using the phone as a tool for navigation, finding information, coordinating arrangements, etc. tends to associate to positive outcomes [21].

That smartphones impact our behavior and well-being—is well documented. But does the world around us also impact our smartphone usage? Some researchers have indeed argued that our experiences in the physical world and contextual factors shape the use of smartphones for different purposes [22, 23]. While plausible, this hypothesis lacks a solid empirical demonstration, due to the limited availability of logged data that captures both offline and online activities, exacerbated by the fact that self-reported data suffers from biases [24, 25].

In this study, we exploit a longitudinal smartphone app usage dataset from ∼500,000 anonymized users from 22 countries spanning multiple continents and a quasi-experimental design to explore a key question: How does the environment that we live in shape the relationship we have with smartphones? Specifically, we focus on a key feature of the physical environment—the level of urbanization—by studying users that live in predominately urban vs. rural environments. The urban/rural split is highly interesting as it is closely related to digitization [26], education [27, 28], income disparities [29], economic resilience [30], and political opinions both in the industrialized west [31] and beyond [32]. Further, there is a growing interest on the impact of exposure to green spaces, disconnection, and rural idyl on smartphone usage, which our work also informs [33]. Another factor emphasizing the importance of this topic is the new possibility of remote working following the COVID-19 pandemic. These changes have made it possible for people of working age to leave densely populated cities, giving rise to a new wave of urban-to-rural migration [34]. Finally, we note that our focus on the urban/rural split is in part motivated by the practical consideration that—unlike many other environmental aspects—we estimate the urban/rural status of each individual using the 2019 GHS settlement model grid [35].

## Results

### Urban–rural divide in phone usage

**Fig. 1.**
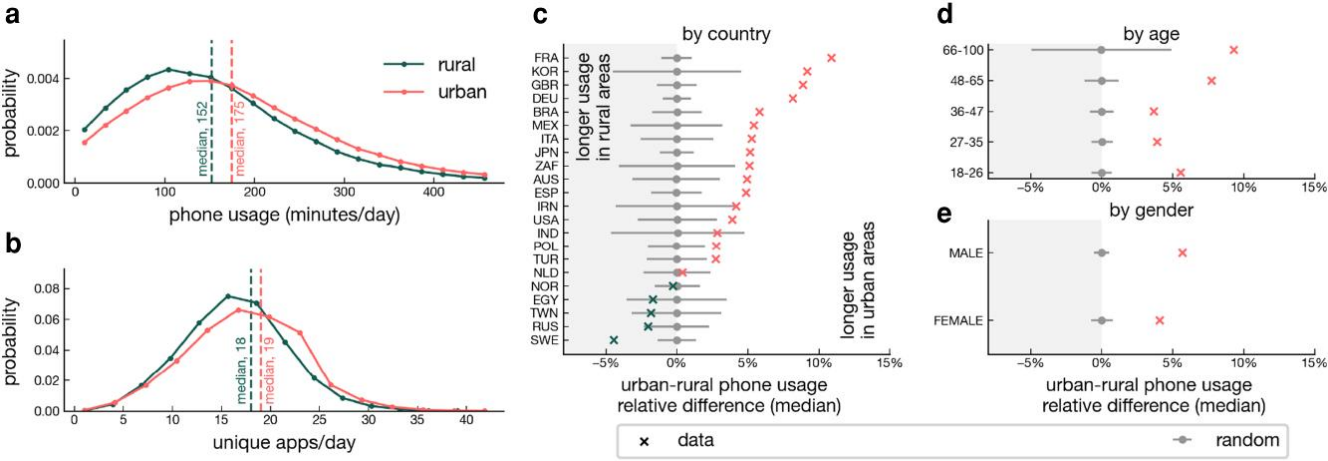
Association between individual characteristics and daily phone usage. a,b) Distribution of a) daily phone usage and b) unique daily apps for rural (dark green filled line) and urban (light red filled line) individuals. Median values are displayed as dashed vertical lines. c) Crosses show the median relative difference in total phone usage across matched pairs of individuals. Results are shown by country of residence, in light red (positive difference) and dark green (negative difference). Dots display the same quantity for randomized data, where individuals in each pair are randomly assigned to the urban or rural group. Errors correspond to standard deviations over 10,000 realizations of the random model. d) Median relative difference in total phone usage across matched pairs of individuals, aggregated by age-group (see description of subplot c). e) Median relative difference in total phone usage across matched pairs of individuals, aggregated by gender (see description of subplot c, errorbars are not visible due to their small size).

We start our investigation of the differences in overall smartphone usage between individuals living in urban versus rural areas. The analyses below are based on a dataset of 464,455 smartphone users, with 324,391 users in the category “urban,” 87,774 in the category “suburban,” and 52,290 in the category “rural.” Individuals were assigned to categories based on their primary residence (see the Materials and Methods section). For each user, we calculate their *median daily phone usage* as follows. First, we find the total time the individual spent using the smartphone on each day of their activity. Second, we computed the median of this quantity across days. Similarly, we compute the *median number of daily apps*, defined as the median number of unique apps per day across all days of the user activity. The distributions of median phone usage and median number of daily apps across users are displayed in Fig. 1a and b. We find that the median daily phone usage across the sample of users under study is 152.2±0.5 min for rural users, and 174.9±0.2 for urban users (see Fig. 1a), where standard errors of the medians are computed by bootstrapping (see Supplementary Section S2). The median number of daily apps is 18±0 for rural users, and 19±0 for urban users (see Fig. 1b).

We first investigate how sociodemographic individual attributes explain the median daily phone usage using a simple linear regression model (see Supplementary Section S2).

In this analysis, our explanatory variables are gender (self-reported by choosing either female or male, see the Materials and methods section for details), age, country of residence (modeled as embedding vectors [36], see Supplementary Fig. S6), and urbanization level (urban, suburban, or rural) of each individual. A linear model explains 14.6% of the variance in the data (see Supplementary Fig. S14a), and all the variables considered are significant, with p≪0.001 (see Supplementary Table S3). The most important feature is age, with phone usage decreasing by 32.7±0.4 min/day per each standard deviation increase in age (see Supplementary Fig. S14b), followed by gender (with females using the phone 15.6±0.7 min/day more than males), country, and urbanization level (with urban individuals using the phone 6.6±1.2 min/day more than rural users). We find that the effect of urbanization on smartphone usage is robust to different formulations of the linear model (see Supplementary Section S3). Note that a feed-forward neural network does not perform significantly better than the linear model ($R^{2}=14.8\%$, see Supplementary Section S2), implying that interactions across features and nonlinearity do not play a strong role. From this preliminary analysis, we conclude that there are significant differences in phone usage between urban and rural individuals. More importantly, this analysis shows that to understand the urban/rural split, we need account for strong phone usage difference driven purely by demographics.

### Probing the urban–rural divide using matching

To understand the key urban/rural split, we now leverage our large dataset to study the phenomena described above, but eliminating the effect of demographic variables using a strategy of matching [37]. We identify pairs (r,u) of individuals in the data, where *r* is a rural individual and *u* is a urban individual, such that *r* and *u* have the same self-reported gender, age-group, and country of residence. We then compute the median relative difference in daily phone usage between urban individuals and their rural counterparts.

Taking into account demographics, we learn that urban individuals use the phone 5.12% more than their rural counterparts ($p\ll 10^{-20}$ by randomization test, see Supplementary Section S2), and use 5.34% more unique apps ($p\ll 10^{-20}$).

Stratifying by country (see Fig. 1c), we observe that the relative difference in phone usage across the rural/urban groups is not equally pronounced across all the countries under study, ranging from −5.26% in Sweden to +11.84% in France. Daily phone usage is statistically higher for urban individuals in 14 countries, for rural individuals in one country (Sweden), and the difference is nonsignificant in 7 countries (see Fig. 1c). The daily number of unique apps used is statistically larger for urban individuals in 15 countries, for rural individuals in three countries (Brazil, Turkey and Sweden), and the difference is nonsignificant in 4 countries (see Supplementary Fig. S15a). We further find that daily phone usage and number of unique apps are higher for urban individuals compared to rural individuals in all age-groups (see Supplementary Fig. S15b) and self-reported genders (see Supplementary Fig. S15c). The results above are robust when urban/rural pairs also matched based on smartphone brand and model (see Supplementary Section S3). Matching on brand ensures that the observed differences are not related to the technology underlying the smartphone devices between the two groups, and suggests that socioeconomic gaps which could be expressed through choice of phone model are also not driving the observed effects.

### Urban–rural divide by apps and app categories

To gain a deeper understanding of differences in urban/rural smartphone usage, we now go beyond the volume of usage and investigate individual usage patterns. For each individual, we compute the fraction of the total smartphone time allocated to different uses. We focus on two different dimensions of smartphone usage: (1) *categories* of applications as defined by the Google Play Store (e.g. Social, Browsing, Business) and (2) *specific applications* (e.g. Facebook, Google Maps, WhatsApp).

**Fig. 2.**
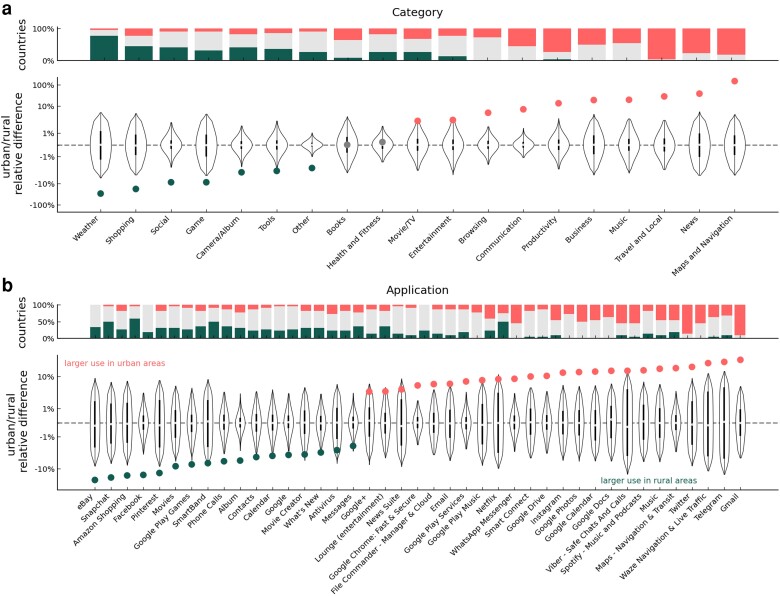
Urban/rural differences in allocation of smartphone usage. Relative difference between urban and rural individuals in fraction of smartphone time by category of application a) and single applications b). In each subplot, the bottom plot shows the distribution of the median difference in randomized pairs (violin plots), and the actual median difference (dots). Dots are colored in light red when the difference is significantly positive (larger usage in urban individuals), in dark green when the difference is significantly negative (larger usage in rural individuals), and in gray elsewhere. The top plot shows the fraction of countries such that usage is significantly larger in urban (light red bars) and rural (dark green bars) individuals. The fraction of countries with nonsignificant difference is displayed in gray. In panel b), we display only the subset among the selected applications such that the median urban/rural difference is significantly different than 0.

Once again, we use the matching as above to eliminate demographic effects. Considering usage patterns, we find substantial differences across urban/rural individuals with respect to all the dimensions of uses under study (see Fig. 2). Focusing on the analysis by app-category, we observe that individuals living in rural areas dedicate a larger fraction of time to use apps categorized as Weather (+29.9%), Shopping (+18.3%), Social (+8.8%), Game (+8.8%), Camera/Album (+3.0%), and Tools (+2.6%), with $p\ll 10^{-20}$. Individuals in urban areas allocate a larger fraction of time to apps categorized as Maps and Navigation (+150.0%), News (+38.7%), Travel and Local (+28.7%), Music (+20.0%), Business (+19.3%), Productivity (+13.7%), Communication (+7.2%), and Browsing (+4.9%), with $p\ll 10^{-20}$. Further, we focus on the total amount of smartphone time by category of apps. Here, we find that not only the fraction of time but also the total time is greater for rural individuals with respect to categories such as Weather (+26.1%), Shopping (+14.1%), Social (+7.5%), and Game (+5.8%, see Supplementary Section S3). In the Supplementary Section S4, we further show the results for a a higher level of app aggregation: *instrumental* vs. *recreational* apps [38].

Finally, in terms of single applications, we note that individuals in rural areas use social media such as Facebook (+17.8%) and Snapchat (+22.5) more, while urban individuals spend a larger fraction of time on Instagram (+14.3) and Twitter (+24.2). Again, results are robust when controlling for the smartphone brand and model (see Supplementary Section S3), as well as total usage (see Supplementary Section S3). Stratifying the data by country reveals that the findings are generally consistent across countries, with some level of variation (see Fig. 2, barplots).

### Urban–rural usage across the day

To further understand differences across residential contexts, we study the allocation of smartphone usage across days of the week and hours of the day. For each week and each individual, we compute the fraction of total smartphone time by day of the week. For each week, we again match rural individuals to their urban counterparts as above, and we compute the relative difference in time allocation across days.

**Fig. 3.**
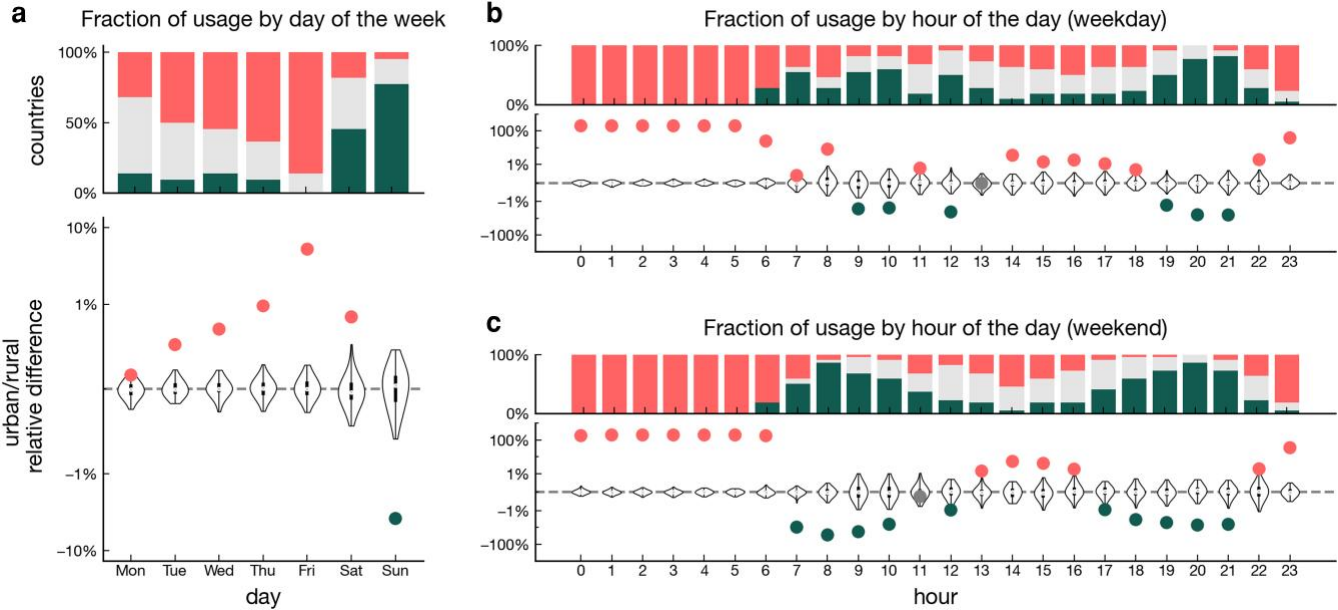
Urban/rural difference across times of the day. Relative difference between urban and rural individuals in fraction of smartphone time for different a) days of the week, and hours of the day during weekdays b) and weekends c). For further explanation of the figure elements, refer to the caption of Fig. 2.

We find substantial differences between urban/rural pairs in the allocation of smartphone time across weekdays (see Fig. 3a, bottom). Individuals living in urban areas use a significantly greater fraction of time on their smartphone on Tuesdays (+0.5%), Wednesdays (+0.7%), Thursdays (+1.0%), Fridays (+5.2%), and Saturdays (+0.8%), while individual living in rural areas tend to use their smartphones more on Sundays (+3.8%). Result are overall robust when we stratify by country (see Fig. 3a, top). We perform a similar analysis at the daily level, and find distinct allocation of smartphone time across hours of the day during weekdays (see Fig. 3b) and weekends (see Fig. 3c). Overall, urban individuals typically dedicate more time to smartphone than their rural counterparts during the night (in the period between 10 PM and 6 AM), as well as in the middle of the day (2 PM to 6 PM on weekdays and 1 PM to 4 PM on weekends). Rural individuals are instead more active during the morning (9 AM to 10 AM on weekdays and 7 AM to 10 AM on weekends) and in the evenings (7 PM to 9 PM on weekdays and 5 PM to 9 PM on weekends).

### Urban/rural contexts causally regulate total smartphone usage

The observation that urban and rural individuals have different smartphone usage patterns has at least two possible explanations: (1) there are inherent differences between individuals who choose to live in urban vs rural areas, and these differences are reflected in smartphone behavior and (2) exposure to urban and rural contexts regulates smartphone behavior (this could happen for example through creating needs for diversion, or offering limited access to resources).

In this section, we test the following hypothesis ($H_{0}$): the observation that urban individuals use smartphones more than their rural counterparts is mainly due to aspect (2) above. An alternative hypothesis ($H_{a}$) is that the observed difference is due to the effect of (1). To test hypothesis $H_{0}$, we study within-individual changes in total smartphone usage for individuals who move their home location during our study period [39–41]. We focus on individuals who move from a urban to a rural area of the same country, and vice versa. Specifically, we consider users for whom we have data in the 36 weeks preceding and following their residential move, and who use the phone at least once in 50% of the days within this period of time, but our results are robust to the specific filtering (see Supplementary Section S3). The selected subset consists of 4,697 residential movers.

**Fig. 4.**
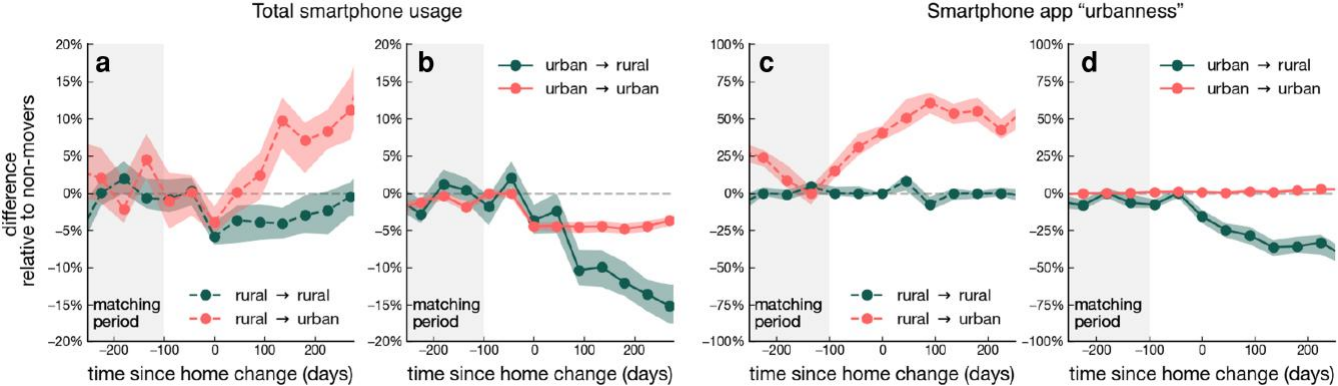
Evolution of smartphone usage for residential movers. Relative median difference in daily phone usage a,b) and urbanness of apps used c,d) between residential movers and their nonmovers counterpart. Results are shown for a–c) individuals moving from rural areas to urban (light red dashed line) or to rural (dark green dashed line) areas; and b–d) individuals moving from urban to rural (dark green plain line) or urban (light red plain line) areas. The line corresponding to no difference relative to nonmovers is shown as a gray dashed line. The period considered to match residential movers to their nonmovers counterpart is displayed as a gray shaded area. Errorbars correspond to 90% confidence intervals computed by bootstrapping.

To study the effect of moving, we take into account different aspects that could impact the way individuals use smartphones, such as time, country of residence and user demographics. We use the matching technique in Ref. [42] for this purpose. We match each residential mover with all individuals that have the same urbanization level (preceding the move), self-reported gender, age-group, and median daily phone usage (in the period between 36 and 14 weeks before the move, ±15 min). Note that—by matching individuals who are active at the same period of time within the same country—we are controlling for both time and country effects. For any given day in the 36 weeks preceding and following the move, we compute the median relative difference in daily phone usage between residential movers and their “nonmovers” counterparts (the baseline). We find that residential moves impact total smartphone total usage. In particular, in the period included between 14 and 36 weeks following the move, the median daily phone usage for individuals moving from urban to rural areas is 14.7% (90% CI: 14.1%, 15.5%) lower than baseline; for individuals moving from urban to other urban areas it is 5.5% (90% CI: 5.4%, 5.6%) lower than baseline; for individuals moving from rural to urban areas it is 11.7% (90% CI: 10.5%, 12.9%) higher than baseline; and for individuals moving from rural to other rural areas is 6.1% (90% CI: 5.3%, 6.7%) lower than baseline (see Fig. 4a and b). Note that, by design, the median daily phone usage for all groups is consistent with baseline in the period included between 36 and 14 weeks preceding the move.

In summary, we observed that, for both urban and rural individuals, a change of context (from urban to rural or vice versa) is associated to a change in total smartphone usage. Thus, our analysis leads to reject hypothesis $H_{a}$ in favor of hypothesis $H_{0}$, showing that exposure to urban and rural contexts regulates an individual’s total smartphone usage.

### Urban and rural contexts causally regulate *how* individuals use their phone

In the sections above, we have found that urban and rural individuals use widely different smartphone applications (see Fig. 3). Here, we test the hypothesis ($H_{1}$) that these differences partly emerge because urban and rural contexts regulate the way we use smartphones for different purposes (aspect (ii) in the section above). An alternative hypothesis ($H_{a}$) is that this result is entirely driven by inherent differences between individuals who choose to live in urban vs rural areas (aspect (i) above). As above, we test hypothesis $H_{1}$ through studying changes in smartphone usage for individuals who change home location. For each smartphone application in the dataset, we estimate its *urbanness*, defined as their relative adoption in urban areas compared to rural areas. Thus, urbanness captures which apps are highly used in urban areas and not used rural areas and vice versa (see Supplementary Section S2 for the precise definition). We compute the *daily smartphone urbanness* for a given individual as the average urbanness of the apps they opened on a given day. We then study the evolution of daily smartphone urbanness for individuals who experience a residential move.

As above, we match each individual with all individuals that have the same urbanization level (preceding the move), self-reported gender, age-group, and median urbanness (in the period between 36 and 14 weeks before the move, ±1%). Further, we only consider matches that are active in the same period of time and country where the move happens, thus controlling for possible effects related to country and time. For any given day in the 36 weeks preceding and following the move, we compute the median relative difference in urbanness between residential movers and their “nonmovers” counterparts (the baseline). Again, we find that residential moves impact smartphone usage patterns. Specifically, we observe that in the period included between 14 and 36 weeks following the move, the smartphone urbanness for individuals moving from rural to urban areas is 52.3% (90% CI: 48.0%, 55.6%) higher than baseline; for individuals moving from urban to rural areas it is 37.0% (90% CI: 34.3%, 39.3%) lower than baseline (see Fig. 4c and d).

Again, we find that, for individuals experiencing a residential move, a change of context (from urban to rural or vice versa) is followed by a change in how smartphones are used. Thus, our analysis leads to reject hypothesis $H_{a}$ in favor of hypothesis $H_{1}$, suggesting that exposure to urban and rural contexts causally regulates how individuals allocate their smartphone usage across different apps.

## Discussion

Drawing on a large-scale dataset of smartphone usage from about 500,000 users from multiple countries across different continents, we demonstrated that the environment an individual lives in shapes how they interact with their smartphone. We found variability in smartphone usage across demographics, and provided novel evidence of the effect of residential environment. Among the demographic features considered, we found that age and gender have the higher association with smartphone usage, confirming that smartphones are more used in younger populations, and by users that identify as female [43, 44]. With respect to the urban/rural split, we confirmed findings from previous small-scale studies, showing that the use of smartphones in rural areas is lower than in more urbanized areas [45, 46]. However, we showed that the higher smartphone usage in urban areas is only true for a certain subset of mobile applications. We found higher use of smartphones in rural environments for activities related to entertainment such as gaming and the use of social apps [47, 48]. In contrast, individuals rooted in urban areas tend to use smartphone more for other purposes, e.g. Communication, Navigation, Travel, Business, and Productivity apps [48, 49]. The observation of rural users using the smartphone for entertainment is supported by the characteristic patterns of usage across the week, with rural individuals using their smartphone more during weekends. Additionally—in the countryside—individuals could be more engaged in occupations such as farming, factory work, or mining, where cell phone use may not be necessary or feasible during weekdays.

Focusing on within-individual changes, we found that moving from a urban to a rural environment (or the other way around) impacts smartphone usage according to the patterns observed above, thus confirming the key role played by the environment for smartphone usage.

Our findings are congruent with the stream of literature on social capital across urban/rural contexts [50]. It has been shown that individuals who live in rural areas tend to value close relationships [45] and have fewer friends who live in their local area [45], rendering the use of communication apps (e.g. for coordinating physical meetings) less necessary. Instead, beyond connecting with friends, social network apps are used for a wide variety of purposes, including entertainment and information seeking [51, 52]. The finding that rural individuals use smartphone more for seeking entertainment can be understood in light of the limited opportunities and access to services experienced in rural areas [53]. For example, it was reported that individuals who live in rural areas devote less time to physical activity for recreation [54], also due to limited access to facilities [55].

Our work has some limitations. First, although our statistical matching technique has enabled us to account for confounding factors, such as age, gender, country, and rural/urban residence, we did not explicitly consider other variables such as economic background and occupation. Second, the interpretation of the results of our quasi-experimental design—focusing on changes in screen time for residential movers—are subject to two assumptions: (i) the results obtained for residential movers can be generalized to other smartphone users, and (ii) common causes underlying changes in residential moves and in phone usage can be neglected. Here, we list some reasons why these assumptions are reasonable. To begin, the subset of residential movers is similar to other users with respect to the distribution of gender, age, country and smartphone time usage (see Supplementary Fig. S2). Next, the results obtained for residential movers and for the whole sample are aligned—both showing a 5 to 10% higher smartphone usage for urban users. Further, the impact of moving on smartphone usage is invariant across diverse geographical and demographic settings [56]. Last, our findings are not due to the residential moves per se, because we do not see any change in patterns of smartphone usage for people moving within the city or within the countryside (see Fig. 4c and d). Finally, due to potential unobserved factors associated with phone ownership, level of education, and self-reported age and gender, our sample population may not be representative of the wider population. However, many of our results are in line with existing literature on smartphone behaviors, suggesting that our novel findings could generalize to wider populations. Nevertheless, understanding the role played by factors beyond the ones addressed in this study could be an interesting avenue for future work [57].

Taken together, our results provide a key example of the environment people live in impacts their smartphone usage patterns. In particular, in rural areas with limited access to services, smartphones are used to entertain users. In areas featuring a variety of opportunities smartphones are used more to facilitate access to services. Our work represents a step towards understanding how our experiences in the real-world shape our relationship with technology. As such, it could open new opportunities towards studying the interplay between online and offline behavior. For example, our findings open the possibility of reinforcing feedback loop effects. One could imagine, for example, that an existing political divide between urban and rural dwellers [31, 32] could be exacerbated by rural individuals spending increasing amounts of time on filter-bubble prone social media platforms [58, 59]. In this sense our work suggests new mechanisms to explain widely documented urban/rural divide in political and socioeconomic attitudes [26, 30, 31]. Overall a deeper understanding of the interplay between environment and smartphone usage will be key to design interventions and technologies that promote mental and physical well-being.

## Materials and methods

### Ethical statement

Our analyses are based on a large-scale mobile-phone dataset collected by a global smartphone and electronics company between 2015 and 2019. All data analysis was carried out in accordance with the European Union’s General Data Protection Regulation 2016/679 (GDPR) and the regulations set out by the Danish Data Protection Agency. We note that, in Denmark, an approval by an ethical committee is not necessary when the work is in accordance with GDPR and the Danish Data Protection Agency regulations. Denmark does not have an IRB system as such, but only Biomedical Research Ethics committees, who will not consider research unless the research involves medical interventions or biological material, see for example http://www.eurecnet.org/information/denmark.html#:˜:text=The%20National%20committee%20(the%20Danish,related%20to%20the%20approval%20of

### Data description and preprocessing

#### Application usage data

The data contains phone usage aggregated by day and app category for ∼4,900,000 Android smartphones users, extracted through a smartphone app. We selected 464,455 individuals using the following filtering criteria. We consider users with at least 20 days of data and such that their median daily phone usage is included between 10 min and 8 h. We filter users from countries with at least 5,000 individuals. After filtering, the data includes users from the following countries: Brasil (BRA), Germany (DEU), Egypt (EGY), Spain (ESP), France (FRA), Great Britain (GBR), India (IND), Ireland (IRN), Italy (ITA), Japan (JPN), Netherlands (NLD), Norway (NOR), Republic of Korea (KOR), Mexico (MEX), Poland (POL), Russia (RUS), Sweden (SWE), Turkey (TUR), Taiwan (TWN), United States of America (USA), South Africa (ZAF). See the breakdown of number of users and smartphone use per country in Table 1.

**Table 1.** Country descriptions.

Country	N. users	Median phone use	Mean phone use ± SE
AUS	5,525	164.53	175.42±1.26
BRA	29,155	203.28	211.93±0.54
DEU	35,887	139.45	154.69±0.49
EGY	5,215	216.13	222.73±1.32
ESP	15,683	151.60	165.80±0.74
FRA	26,864	153.01	164.93±0.56
GBR	34,061	164.57	176.13±0.52
IND	10,974	202.03	209.83±0.88
IRN	6,756	215.15	222.67±1.16
ITA	6,600	149.53	163.09±1.11
JPN	153,726	156.88	173.45±0.27
KOR	6,949	199.83	210.58±1.28
MEX	11,591	213.37	220.60±0.90
NLD	7,988	159.18	171.47±1.01
NOR	5,185	142.68	153.93±1.19
POL	9,968	143.71	157.02±0.89
RUS	22,589	174.62	186.04±0.67
SWE	13,716	141.85	154.91±0.76
TUR	20,488	209.83	218.36±0.65
TWN	22,096	210.37	219.56±0.73
USA	8,330	188.44	199.55±1.12
ZAF	5,109	174.92	186.08±1.27

Number of individuals and smartphone use (in minutes) by country.

#### Metadata

Users self-reported their age and gender at the time of registration. Individuals are aged between 18 and 100 years old, with an average age of 36 years. In some of the analyses, we grouped user by age-group, where we considered the following age groups: 18–26, 27–36, 36–48, 48–66, 66+. Gender is self-reported by users, who could choose between the options male and female at time of registration. About one-third of individuals identified as female in the dataset. Figure S1 in the Supplementary material shows the number of individuals in our data divided by self-reported gender (Supplementary Fig. S1b) and age group (Supplementary Fig. S1c).

#### Urbanization and country of residence data

The level of urbanization surrounding users home location was estimated using location data collected via the smartphone. Location data was preprocessed using the Infostop algorithm [60]. The home location was identified as the stop where users spent most of their time between 9 PM and 6 AM, over a sliding window of 28 days. We estimated an individual’s country of residence from the coordinates of the location that is identified as the user’s home location for the longest period of time. The urbanization level around users home locations was estimated using the 2019 GHS settlement model grid with 1 km resolution [35]. The GHS model classifies settlement typologies into urban, suburban, and rural, via a logic of population size, population, and builtup area. Table 2 shows the number of individuals in our data and smartphone use divided settlement typologies. Note that we only consider the rural and urban types in the analysis.

**Table 2.** Urbanization description.

Urbanization	N. users	Median phone use	Mean phone use ± SE
Rural	52,290	152.07	165.75±0.42
Suburban	87,774	160.43	173.73±0.33
Urban	324,391	174.52	186.63±0.18

Number of individuals and smartphone use (in minutes) by urbanization level.

## Supplementary Material

pgad357_Supplementary_Data

## Data Availability

Data that support the findings of this study will be made available upon publication.
