# Supplementary material for: Exposure to urban and rural contexts shapes smartphone usage behavior
Source: PNAS Nexus. 2023 Nov 28;2(11):pgad357. doi: 10.1093/pnasnexus/pgad357 (PMC10683949; doi:10.1093/pnasnexus/pgad357)
Supplement: pgad357_Supplementary_Data [file pgad357_supplementary_data.pdf]

Supplementary Material

S1. Data description

Distribution of demographics. The distribution of users across countries, genders, age group and urbanization levels is shown in Fig. S1. In Fig. S2, we show the distribution of the subset of residential movers, which display comparable characteristics.

Smartphone application categories. We used categories as defined by the Google Play Store, the official app store for certified devices running on the Android operating system. The distribution of usage by category is shown in Fig. S4. To better understand the app categorization, we show three examples of apps from each category of interest in Table S2. We selected categories used by at least 40% of users in all the countries under study. The selected categories are: Books, Browsing, Business, Camera/Album, Communication, Entertainment, Game, Health and Fitness, Maps and Navigation, Movie/TV, Music, News, Productivity, Shopping, Social, Tools, Travel and Local, Weather. The distribution of usage and fraction of time by category are respectively shown in Supplementary Information Figure S3 and S4. For additional results, app categories are then further categorized into "Recreational" and "Instrumental" based on the findings in [1] (see Table 3 in the article). In the article [1], the authors ran an experiment where they asked individuals for what purpose they would use different apps. Then, they identified categories as "Instrumental" if they are used for a significantly larger fraction of time towards goal-directed and purposeful actions, while they categorize as "Ritualistic" (here Recreational) if they mostly serve habitual and diversionary use (see Table S1).

Category	Type
Books	Recreational
Browsing	Recreational
Business	
Photography	
Communication	
Entertainment	
Game	Recreational
Health and Fitness	Instrumental
Maps and Navigation	Instrumental
Movie/TV	
Music	
News	Recreational
Productivity	Instrumental
Shopping	
Social	Recreational
Tools	Instrumental
Travel and Local	Instrumental
Weather	Instrumental

Table S1. Instrumental and Recreational apps. Apps are categories into "Instrumental" and "Recreational" based on their category. Our categorization is based on the results in [1]. Note that some categories are not distinctively Recreational or Instrumental.

Selection of apps. The analysis at the app level (see Figure 2) is run considering only a subset of apps. We focused on apps that are widely used either within the rural or the urban sample. The selection was done as follows. We stratified users by gender, age-group, urbanization and country of residence, thus obtaining 660 groups (we have 22 countries, 2 genders, 3 urbanization groups, and 5 age groups). We considered the subset of apps that are used by at least 50% of users in at least 10 rural groups or in 10 urban groups. Finally, we remove apps that correspond to pre-installed system packages. We obtain a list of 81 apps. Note that, in the analysis reported in Fig. 2, for each application, we discarded users that do not use the app at all. This is to account for the fact that, in many cases, applications are used by less than 50% of users, implying that the median difference across pairs of individuals is 0.

Category	Apps
Books	Audiobooks from Audible, Wikipedia, Amazon Kindle
Browsing	Chrome Browser - Google, Firefox Browser fast & private, Phone Browser
Business	OfficeSuite + PDF Editor, File Commander - File Manager, Facebook Pages Manager
Communication	WhatsApp, Phone Messaging, Phone Calls
Entertainment	Google Play Games, PlayStation®App, Steam
Game	Pokémon GO, Clash of Clans, Clash Royale
Health and Fitness	SmartBand, Bluelight Filter, Google Fit
Maps and Navigation	Uber, Grab, NAVITIME - Map & Transfer Navi
Move and TV	YouTube, Movies, Netflix
Music	Phone Music, Spotify Music, Google Play Music
News and Magazines	SmartNews, Google News, feedly: your work newsfeed
Photography	Phone Album, Google Photos, QuickPic Gallery
Productivity	Phone Calendar, Google Drive, Microsoft Outlook
Shopping	Amazon Shopping, eBay, Wish
Social	Facebook, Instagram, Snapchat
Tools	Google, Google Play services, Antivirus
Travel and Local	Maps, Navigation & Transit, Airbnb, TripAdvisor
Weather	Phone Weather, Yahoo Weather, Weather & Clock Widget

Table S2. Apps in categories. Examples of apps that can be found in the categories of interest.

S2. Details on the methodology.

Definition of urbanness. We define the *urbanness* of a smartphone application as follows. For any socio-demographic group of users with given gender, age-group, and country, we compute the quantity $u = 2 \cdot 100 \cdot (N_u - N_r)/(N_u + N_r)$, where N_u and N_r are the number of users from urban and rural areas, respectively. The median urbanness by smartphone app category is shown in Supplementary Information Section S4.

Estimating the standard error of the median by bootstrapping. The standard error of the median is estimated using the bootstrap method. We note that the method is applied to the vector of data-points where each point is the median daily smartphone usage (or the number of apps opened daily) computed for one individual across days. The method can be summarized as follows:

1. Randomly sample with replacement N datapoints from the available data. This is called a bootstrap sample.
2. Calculate the median of the bootstrap sample
3. Repeat steps 1 and 2 B times and then calculate the standard deviation of the B medians. This is the bootstrap estimate of the standard error the median.

Unless specified, we take N to be equal to the size of the available data, and $B = 100,000$.

Country embeddings. In order to include individuals' country of residence as a feature of the model capturing smartphone usage, we have to convert categorical data representing countries to numerical values. To this end, we use an entity embedding approach [3], as we will explain in the following of this section. Compared to one-hot encoding, the entity embedding approach has two advantages: (i) it results in a fixed

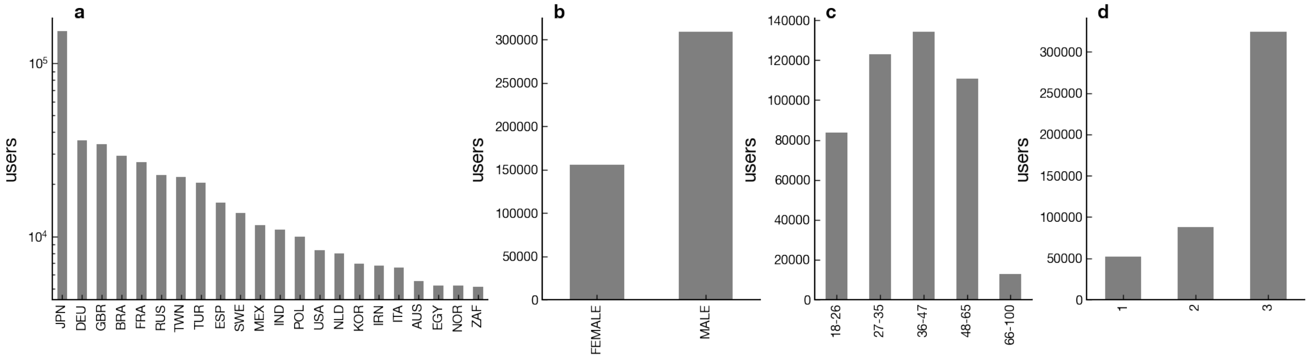

Fig. S1. Data Description. Number of users by country (**a**), gender (**b**), age group (**c**) and urbanization level (**d**), where 1 refers to rural, 2 to suburban, and 3 to urban areas.

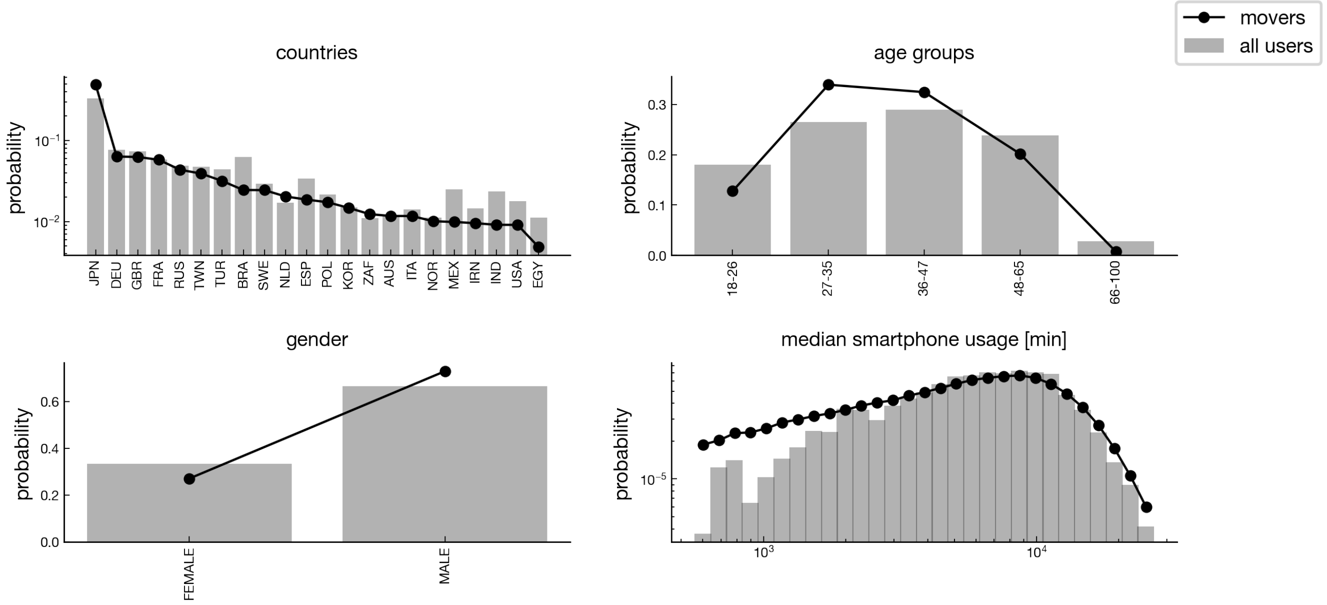

Fig. S2. Comparison between the distribution of movers and all users. Distribution of residential movers (black line) and all users (gray bars) by country (top left), gender (bottom left), age group (top right) and median smartphone usage (bottom right).

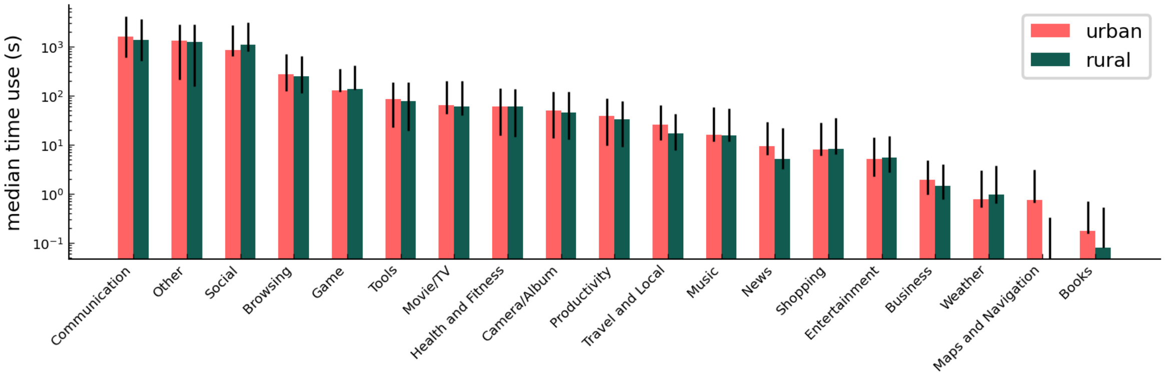

Fig. S3. Distribution of smartphone usage across categories. Median time (in log scale) spent by category for the application categories considered for urban (red bars) and rural (green bars) individuals. Medians are obtained stratifying by country, age, and gender. Error-bars correspond to the middle interquartile range.

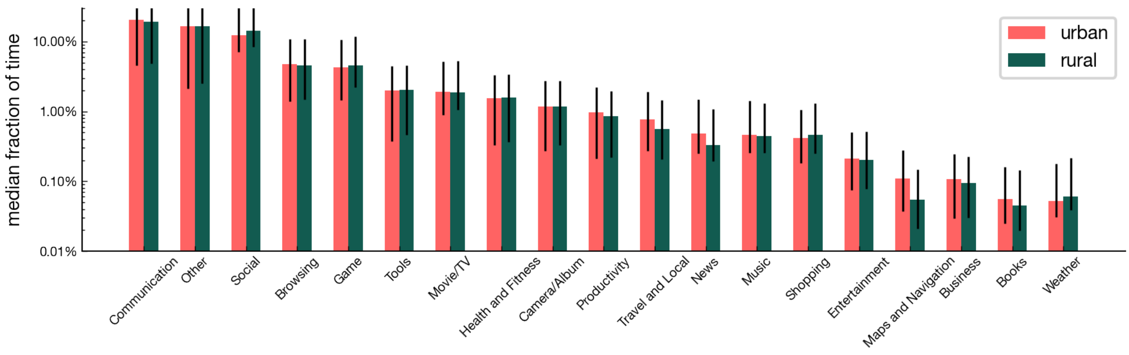

Fig. S4. Distribution of smartphone usage across categories. Median fraction of time spent by category for the application categories considered for urban (red bars) and rural (green bars) individuals. Medians are obtained stratifying by country, age, and gender. Error-bars correspond to the middle interquartile range.

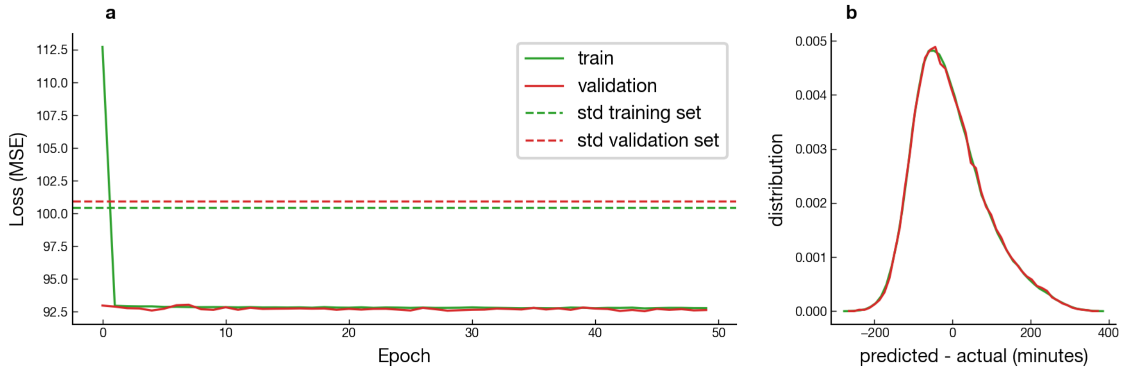

Fig. S5. Learning country embeddings. a) Loss Function (Mean Squared Error) between the predicted phone usage and the actual phone usage over epochs for the train (red line) and validation (green line) sets. The standard error of the train (red dashed line) and validation set (green dashed line) is shown as a reference. **b)** Distribution of the difference between predicted and actual phone usage for the train (green line) and validation (red line) sets.

number of features even if we have a large number of countries and (ii) it enables to capture the relations between the countries [3]. In order to compute the country embeddings, we use a feed-forward neural network with three linear layers, each with 100 nodes, and 10% dropout. The model learns embedding by learning the relation between individuals' median daily phone usage from the following features: gender, country, urbanization level, age. We train the model with 90% of the data for 50 epochs (see Figure S5). The model achieves a coefficient of determination $R^2 = 0.153$ on the train set and $R^2 = 0.148$ on the validation set. In terms of embedding dimension, we explore the range included between 1 and 10, and select a 4-dimensional embeddings, which achieves the maximum R^2 on the validation set. In Fig. S6, we show the Euclidean distance between embedding vectors representing countries. Interestingly, we observe that geographically or culturally close countries (e.g. European countries) tend to lie at smaller embedding distance from each other, implying they are characterized by more similar patterns of smartphone usage.

Linear regression analysis. To identify the factors associated with overall phone usage, we fit a linear regression model that estimates individuals' median daily phone usage from a set of individual features. The features include gender, age, country of residence (modelled as the embedding vectors learned in the previous section, see Supplementary Information Figures S5 and S6), and urbanization level. The linear model is defined as:

$$m_i = \beta_0 + \sum_k \beta_k x_{i,k} + \epsilon_i$$

, where m_i is the median daily phone usage for individual i, β_0 is the regression intercept, β_k is the coefficients associated to each feature k, $x_{i,k}$ is the value taken by feature k for individual i, and ϵ_i is the error for individual i, which is assumed to be normally distributed $\epsilon_i \sim N(0, \sigma^2)$. We used Ordinary Least Squares regression to estimate the coefficients. The results of the linear regression are shown in Fig. S14.

Matching experiment (urban with non-urban individuals). Matching is a statistical technique used to evaluate the effect of a treatment by comparing the treated and the non-treated groups in an observational study. Matching experiments aim at replicating a randomized experiment as closely as possible by obtaining treated and non-treated groups with similar covariate distributions. In this work, we performed a matching experiment for comparing phone usage across the urban and the rural groups [2]. We perform the matching experiment as follows:

1. **Create the set of matched pairs.** For each rural individual r in the dataset, we find all urban individuals

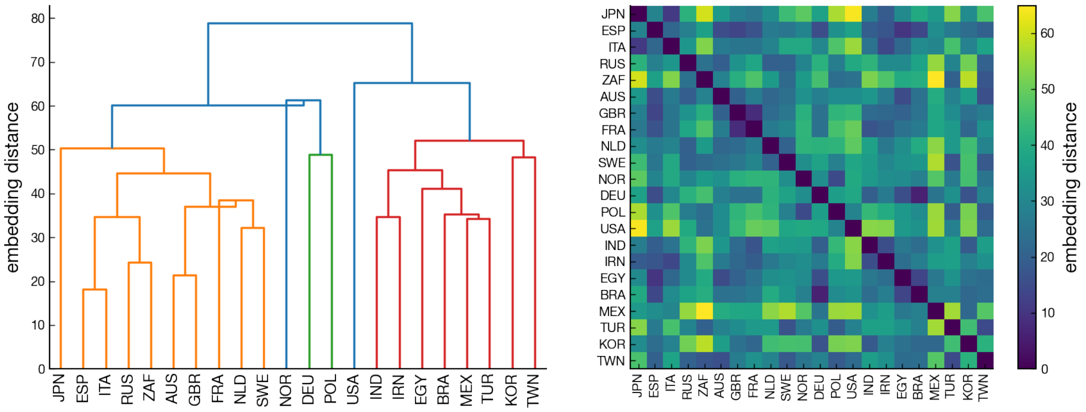

Fig. S6. Country embeddings. left) Dendogram capturing the hierarchical centroid linkage clustering between countries based on their embedding distance (computed as the euclidean distance between embeddings). right) Embedding distance (computed as the euclidean distance) between pairs of countries in the dataset.

u such that r and u have the same gender, nationality, and age-group. Through this process, we create a set of pairs of matched individuals.

2. **Compute the rural/urban divide.** For each pair, we compute the relative difference $d = 2(x_u - x_r)/(x_r + x_u)$, where x_u (x_r) is a quantity of interest computed for the urban (rural) individual in the pair. Then, we first compute the median relative difference across matches (e.g. we find the difference between any rural user and their typical urban counterpart). Finally, we compute the median D across rural individuals.

To assess the significance of the relative difference D between the urban and the rural groups, we perform the following randomization test:

1. **Create the typical match for each user.** For each rural user, we compute the median value of all their urban matches.
2. **Create the set of randomized matched pairs.** For each pair in the set of matched pairs, we shuffle the urbanization labels, such that each individual in the pair is randomly assigned to either the rural or the urban group.
3. **Compute the divide for the randomized groups.** We compute the relative difference between urban and rural users as mentioned in step 2 above.

We repeat the two steps above $N = 10,000$ times. We test the null hypothesis that D is statistically different from 0. We estimate the one-tail p-value p as the fraction of instances in which D_r is more extreme than D. We reject the null hypothesis if $p < \alpha$, where α is the chosen significance level.

Residential movers. An individual's home location is identified as the stop where users spent most of their time between 9AM and 6AM, over a sliding window of 28 days. We identify residential movers as individuals with a single home location in the period included between t_M and $t_M - \Delta t$ and a different single home location in the period included between t_M and $t_M + \Delta t$, where t_M is identified as the moving day. In the main text, we considered $\Delta t = 36$ weeks (see Supplementary Information Section S3 for different choices). We considered only users with smartphone activity in at least 50% of the days included between $t_M - \Delta t$ and $t_M + \Delta t$ (see Supplementary Information Section S3 for different choices).

1. **Create the set of randomized matched pairs.** We matched each residential mover with their non-movers counterparts as follows. For each residential mover i changing home location at time t_M, we found all individuals j such that i and j have the same gender, nationality, and age-group, and such that the median daily smartphone usage m_i of user i in the period included between $t_M - \Delta t$ and $t_M - \delta t$ is within 15 minutes of the median daily smartphone usage m_j of user j ($m_j - 7.5$ minutes $\leq m_i \leq m_j + 7.5$ minutes). We considered $\Delta t = 36$ weeks and $\delta t = 14$ weeks.
2. **Compute the difference between movers and non-movers over time.** For each pair, we compute the relative difference $d = 2(x_i(t) - x_j(t))/(x_i(t) + x_j(t))$, where $x_i(t)$ ($x_j(t)$) is a quantity of interest computed for the mover (non-mover) individual in the pair on any given day t. Then, we first compute the median relative difference across matches (e.g. we find the difference between any mover user and their typical non-mover counterpart). Finally, we compute the median D across residential movers.

S3. Robustness checks

Results are robust under a Mixed-Effect Linear Model for repeated measurements. The linear model presented in the manuscript models the median time usage for each individual. Here we develop a mixed-effect linear model for repeated measurement, where we model the yearly daily median smartphone usage by user. The model can be represented as:

$$
\begin{aligned}
\{\text{Screentime}_{ijt} &= \beta_0 + \beta_1 \times \text{Age}_{ijt} + \beta_2 \times \text{Gender}_i + \\
&+ \sum_{k=1}^{3} \beta_{3k} \times \text{UrbanLevel}_{k,it} + \alpha(\text{CountryYear}_{jt}) + \\
&+ u_j + v_{i(j)} + \epsilon_{ijt}\}
\end{aligned} \tag{1}
$$

Where:

- Screentime_{ijt} is the median daily screen-time for indiviudal i at year t in country j.
- $\beta_0, \beta_1, \beta_2, \beta_{3k}$ are the fixed effects coefficients for the intercept, age, gender, and urban level, respectively.
- $\alpha(\text{CountryYear}_{jt})$ represents the fixed effect for each country-year combination.
- u_j is the random effect for each country j, $u_j \sim \mathcal{N}(0, \sigma_u^2)$.
- $v_{i(j)}$ is the random effect for each user i nested within country j, $v_{i(j)} \sim \mathcal{N}(0, \sigma_v^2)$.
- ϵ_{ijt} is the residual error term, $\epsilon_{ijt} \sim \mathcal{N}(0, \sigma^2)$.

Note: For Gender_i, 0 represents Females and 1 represents Males. For $\text{UrbanLevel}_{k,it}$, 1 indicates that an individual i lives in urbanization level k at time t, with k ranging from 1 for rural to 3 for urban.

Due to the large size of the data, we fit the model to a random sample of 28,960 individuals. The results of the model are presented in Table S4. Under this model, we find that urban individuals, everything else equal, use the smartphone 4.7 ± 1.9 minutes/day more than their rural counterparts.

Results are robust when controlling for smartphone brand and model. We verified that the results presented in the main text hold when individuals are also matched based on the brand and model of their smartphone device. We find that the median relative difference in daily phone usage is 6.5% and it is significantly larger than 0 against the randomization test ($p \leq 10^{-20}$), see Fig. S7. When stratifying by country, the difference is statistically larger than 0 in 13, smaller than 0 in 1 country, and non-significant in 8 countries. The difference is significantly larger than 0 in all age groups and genders. The median relative difference in number of unique apps is 6.45% and it is significantly larger than 0 against the randomization test ($p \leq 10^{-20}$) in 20 countries, in all age-groups and genders (see Fig. S7). In Fig. S9 we show the distribution of usage across categories, apps, and types of usage when controlling by device brand and model. We observe that recreational usage is larger in the rural group (+3.1%), while instrumental usage is larger in the urban group(+3.7%). Categories used predominantly by the rural group are Weather (+29.2%), Shopping (+23.2%), Game (+11.7%), Social (+10.1%), Camera (+4.6%). Categories used predominantly by the urban group are Maps and Navigation (+109.0%), News (+26.8%), Travel and Local (+22.5%), Music (+13.2%), Productivity (+8.9%), Communication (+5.2%) and Browsing (+2.4%). In terms of single applications, we note that individuals living in rural areas use more social media such as Facebook (+19.6%) and Snapchat (+30.5), while urban individuals spend a larger fraction of time on Instagram (+7.4) and Twitter (+14.7).

Results on allocation of smartphone time are robust when controlling for typical total usage. We verified that the results presented in Fig. 2 hold when individuals are matched also based on their typical daily smartphone usage. We categorize users in groups based on their typical usage: less than 1 hour; 1 to 2 hours; 2 to 3 hours; 3 to 4 hours; 4 to 5 hours; 5 to 6 hours; 6 to 7 hours; more than 7 hours. Results obtained when matching by typical usage are shown in S10. We observe that recreational usage is larger in the rural group (+2.9%), while instrumental usage is larger in the urban group(+7.0%). Categories used predominantly by the rural group are Weather (+31.2%), Shopping (+19.9%), Game (+11.5%), Social (+10.0%), Camera (+2.7%). Categories used predominantly by the urban group are Maps and Navigation (+127.0%), News (+37.9%), Travel and Local (+29.5%), Business (+19.7%), Music (+17.8%), Productivity (+14.2%), Communication (+7.9%) and Browsing (+5.3%). In terms of single applications, we note that individuals living in rural areas use more social media such as Facebook (+18.6%) and Snapchat (+24.2), while urban individuals spend a larger fraction of time on Instagram (+12.7) and Twitter (+20.3).

Results on residential moves are robust to changes of the parameters. We verified that the results presented in Figure 4 are robust to changes in the parameters for identifying residential movers. In the main text, we showed the results obtained for users that live in a given location for $\Delta t = 36$ weeks, and with smartphone activity in at least 50% of the days included between $t_M - \Delta t$ and $t_M + \Delta t$. Here, we show the results obtained for $\Delta t = 50$ weeks (Figure S13), and for thresholds of 40% (Figure S12) and 60% (Figure S11).

Results on residential moves are robust under a difference-in-differences model with Two-way fixed effects. To corroborate the results from the methodology using matching to study the effects of the environment on smartphone use through movers, we additionally computed a difference-in-differences with a Two-way fixed effects model defined as follows:

$$SMU_{it} = \sum_{g}^{G} \sum_{t}^{T} \tau_{gt} M_{it} + \gamma_i + \theta_t + \epsilon_{it}, \tag{2}$$

where, SMU_{it} is the smartphone use of the individual i at time t, γ_i is the unit fixed parameter to control for the individual user and thus its characteristics, such as age, country, gender, etc., θ_t is a time fixed parameter, M_{it} is the dummy parameter indicating when the move happens for any individual and τ_{gt} is the treatment effect. Note that here we divided users in G groups, defined by the year in which the move happens. We find an average reduction of smartphone use of -878.55 ± 21.05 seconds for individual moving from urban to rural areas, and an average increase of 210.14 ± 23.44 seconds for users that move from rural to urban areas, thus confirming our hypothesis that the environment shapes smartphone use.

S4. Additional Results

Urban individuals use more apps. The daily number of unique apps used during a day is statistically larger for urban individuals. The results stratified by country, age and gender are presented in Fig. S15.

Urban individuals use more recreational apps. Focusing on the recreational and instrumental use of the smartphone, we observe that individuals living in rural areas allocate a larger fraction of smartphone time on recreational activities (+2.1%), while individuals living in urban areas allocate a larger fraction of smartphone time for instrumental use (+6.0%), with $p \ll 10^{-20}$ (Fig. S16).

Typical usage across types, apps, and categories. We further quantify urban/rural differences in usage by studying the typical daily time allocated to different apps and categories of apps. For any given app i, we compute the an individual's typical daily usage as the product $m_i \cdot f_i$, where m_i is the median time of usage across days in which the app is used at least once, and f_i is the fraction of days in which the app is used at least once. Differences across urban/rural individuals are shown in Fig. S17. We observe that individuals living in urban areas allocate a larger time to recreational

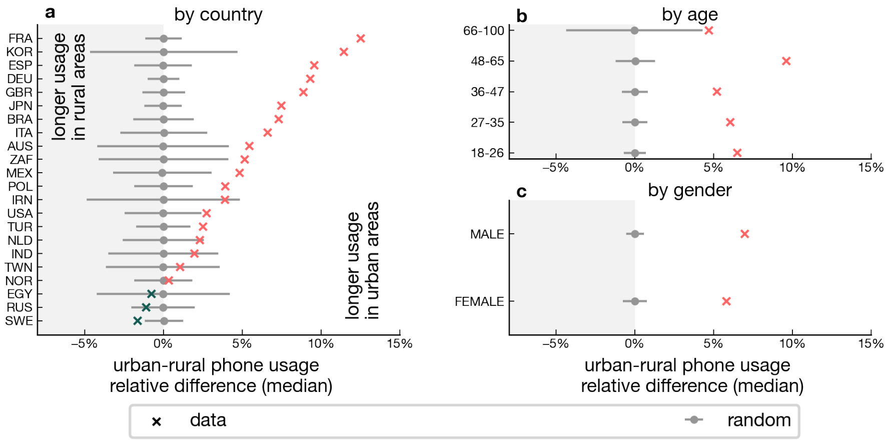

Fig. S7. Rural/Urban differences in phone usage (controlled for device model and brand). a) Crosses show the median relative difference in total phone usage across matched pairs of individuals. Results are shown by country of residence, in red (positive difference) and green (negative difference). Dots display the same quantity for a null model, where individuals in each pair are randomly assigned to the urban or rural group, with the corresponding errors (standard deviations over 10000 realizations of the random model). **b)** Median relative difference in total phone usage across matched pairs of individuals, aggregated by age-group (see description of subplot b). **c)** Median relative difference in total phone usage across matched pairs of individuals, aggregated by gender (see description of subplot b, errorbars are not visible due to their small size).

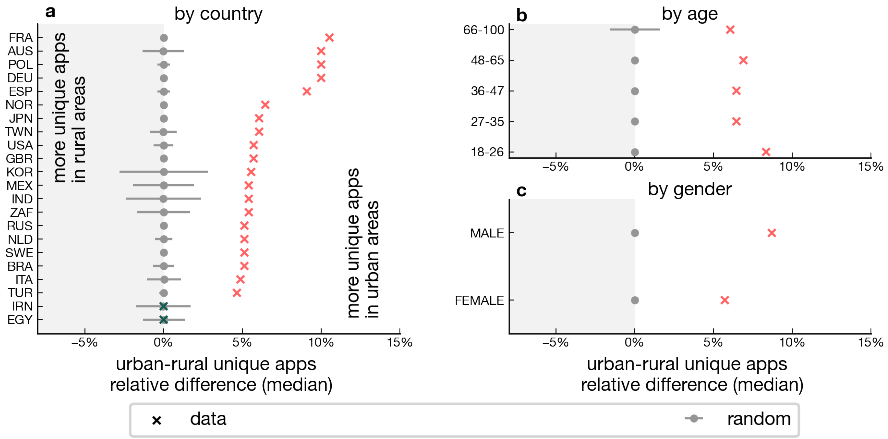

Fig. S8. Rural/Urban differences in number of unique apps (controlled for device model and brand). a) Crosses show the median relative difference in number of unique apps across matched pairs of individuals. Results are shown by country of residence, in red (positive difference) and green (negative difference). Dots display the same quantity for a null model, where individuals in each pair are randomly assigned to the urban or rural group, with the corresponding errors (standard deviations over 10000 realizations of the random model). **b)** Median relative difference innumber of unique apps across matched pairs of individuals, aggregated by age-group (see description of subplot b). **c)** Median relative difference in number of unique apps across matched pairs of individuals, aggregated by gender (see description of subplot b, errorbars are not visible due to their small size).

(+2.2%) and instrumental usage (+16.4%). Individuals living in rural areas allocate a larger time to apps categorized as Weather (+26.1%), Shopping (+14.1%), Social (+7.5%), Game (+5.8%). Individuals living in urban areas allocate a larger time to apps categorized as Maps and Navigation (+172.2%), News (+48.8%), Travel and Local (+42.7%), Music (+25.6%), Business (+20.7%), Productivity (+19.6%), Communication (+14.3%), Browsing (+11.6%), Movie/TV (+7.0%), Entertainment (+6.9%), Health and Fitness (+6.2%), Health and Fitness (+6.2%), Tools (+3.1%).

Median urbanness by category. In figure S18, we show the median urbanness aggregated by smartphone app category. We have discarded apps with less than 20 urban users or less than 20 rural users. For any socio-demographic group of users with given gender, age-group, and country, we compute the quantity $u = 2 \cdot 100 \cdot (N_u - N_r)/(N_u + N_r)$, where N_u and N_r are the number of users from urban and rural areas, respectively.

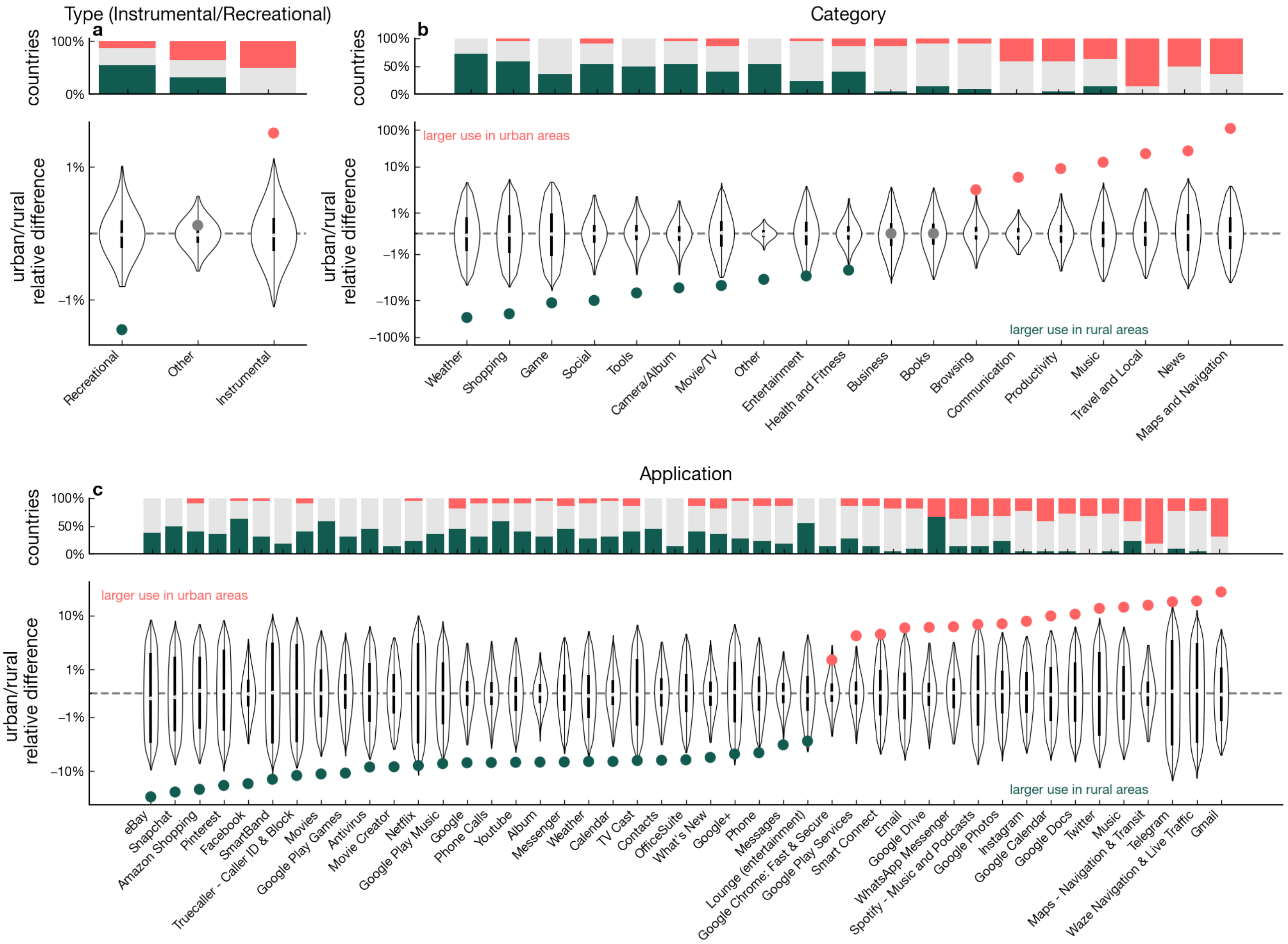

Fig. S9. Urban/rural differences in allocation of smartphone usage (controlled for device model and brand). Relative difference between urban and rural individuals in fraction of usage by type (recreational/instrumental) **(a)**; category of application **(b)**; and single applications **(c)**. Here, users are matched by device model and brand (beyond gender, age group and country). The figure is described in the caption of Fig. 2

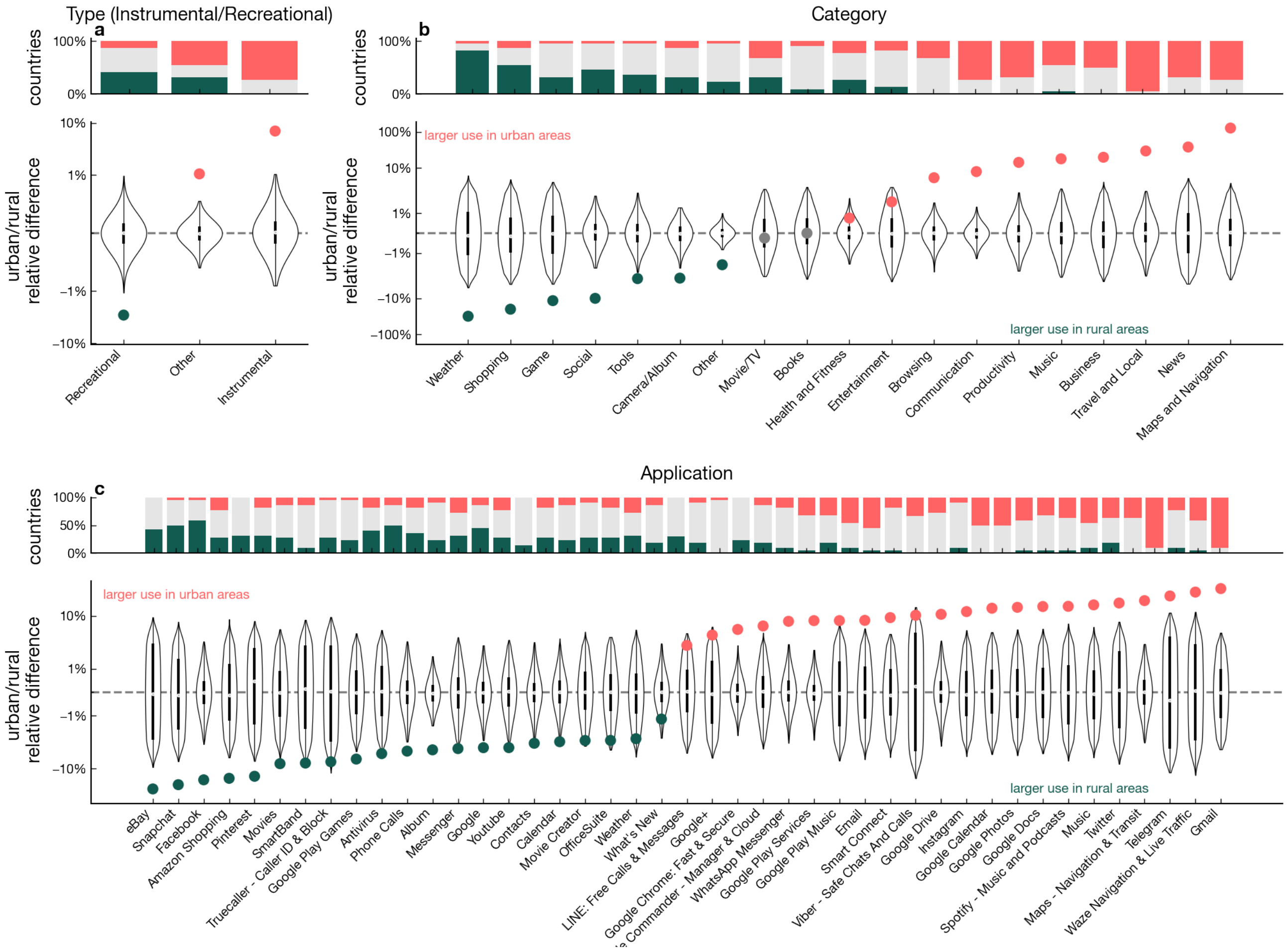

Fig. S10. Urban/rural differences in allocation of smartphone usage (controlled for typical usage). Relative difference between urban and rural individuals in fraction of usage by type (recreational/instrumental) **(a)**; category of application **(b)**; and single applications **(c)**. Here, users are matched by typical daily usage (beyond gender, age group and country). The figure is described in the caption of Fig. 2.

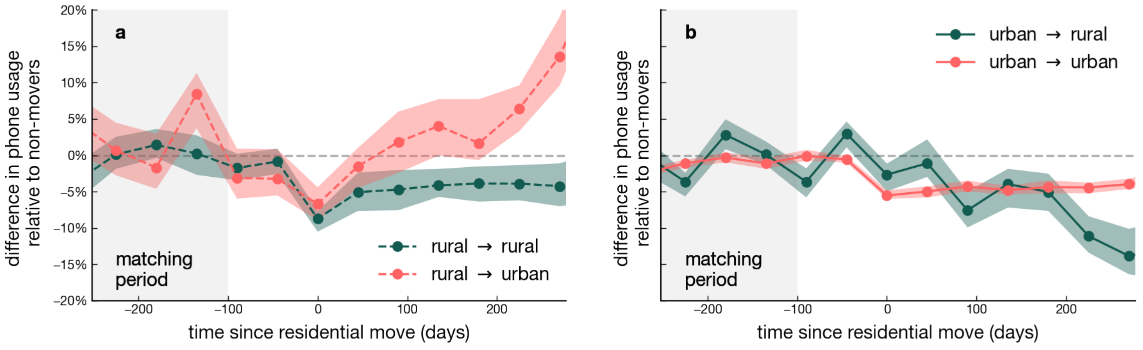

Fig. S11. Evolution of daily phone usage for residential movers. See caption of Figure 4. Results are shown for users that live in a given location for $\Delta t = 36$ weeks, and with smartphone activity in at least 60% of the days included between $t_M - \Delta t$ and $t_M + \Delta t$.

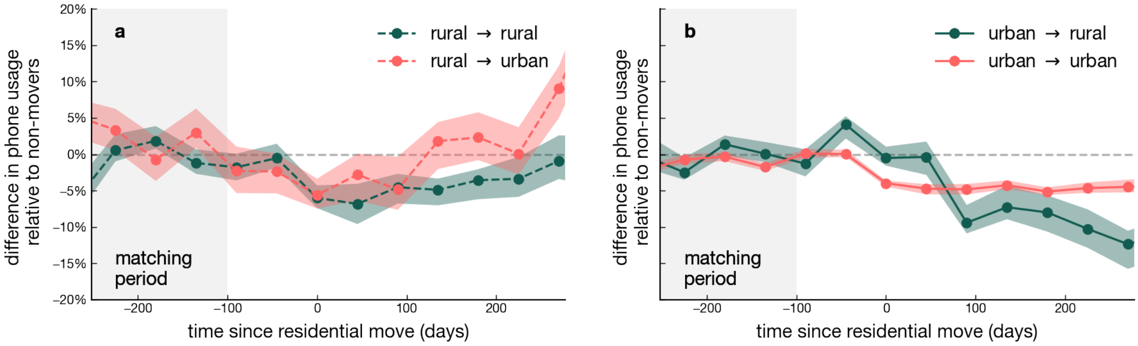

Fig. S12. Evolution of daily phone usage for residential movers. See caption of Figure 4. Results are shown for users that live in a given location for $\Delta t = 36$ weeks, and with smartphone activity in at least 40% of the days included between $t_M - \Delta t$ and $t_M + \Delta t$.

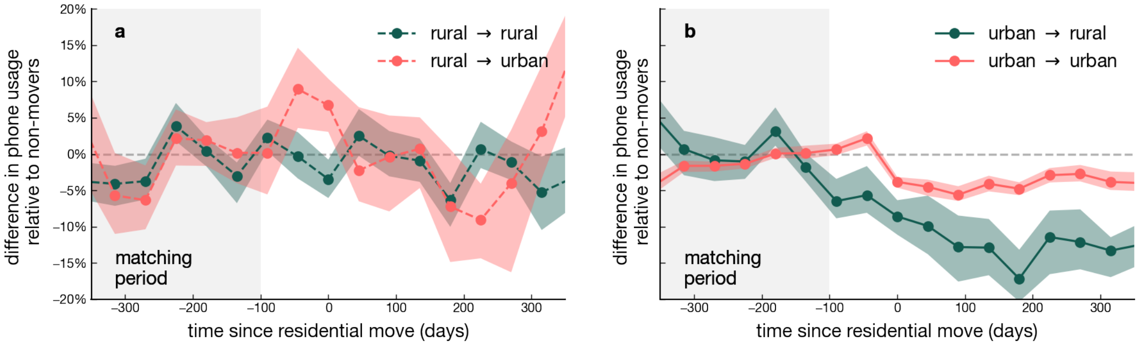

Fig. S13. Evolution of daily phone usage for residential movers. See caption of Figure 4. Results are shown for users that live in a given location for $\Delta t = 50$ weeks, and with smartphone activity in at least 50% of the days included between $t_M - \Delta t$ and $t_M + \Delta t$. Note that we have only 175 movers from rural areas with this selection.

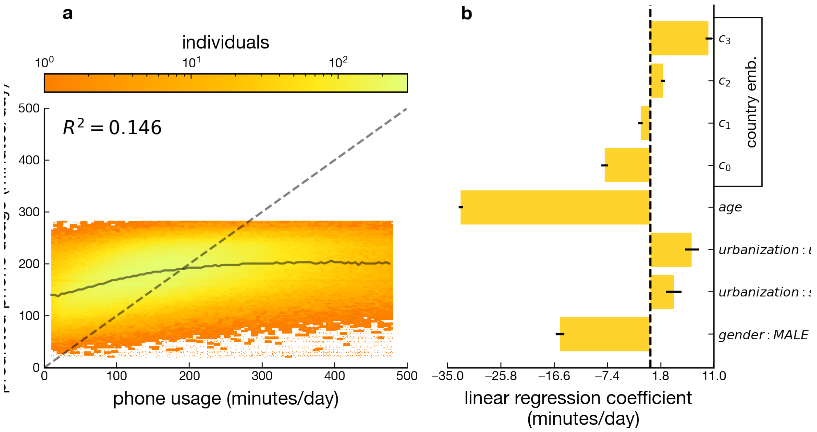

Fig. S14. Explaining differences in median screen time using linear regression. a) Predictions of a linear regression model against empirical values for daily phone usage. The fraction of variance explained by the model is $R^2 = 0.15$. Results are presented as a two dimensional histogram using a heatmap (see colorbar). The dashed black line corresponds to the identity line, the filled black line to the median for each bin along the x-axis. **b)** Coefficients of the linear regression (x-axis) for each of the variables considered (y-axis). c_0, c_1, c_2 and c_3 are the elements of the country embedding vectors. Urbanization and gender are categorical features, with the "rural" and the "female" categories as reference, respectively. Error bars indicate 95% confidence intervals as estimated using ordinary least squares. The full model is introduced in Table S3

.

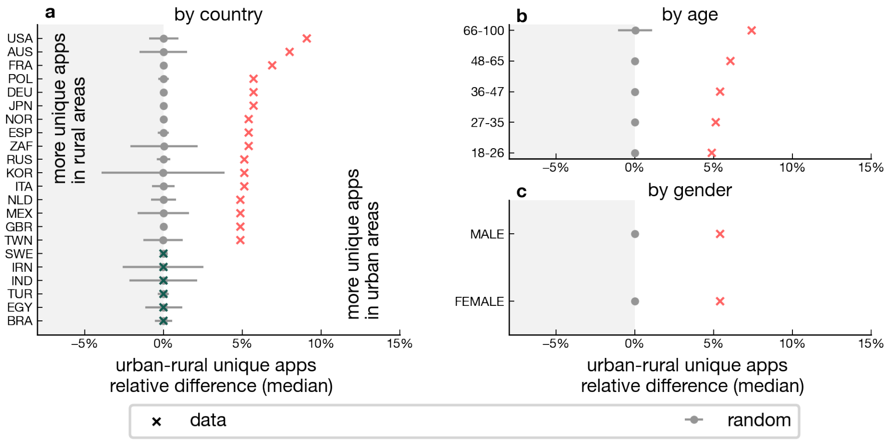

Fig. S15. Rural/Urban differences in number of unique apps. a) Crosses show the median relative difference in number of unique apps across matched pairs of individuals. Results are shown by country of residence, in red (positive difference) and green (negative difference). Dots display the same quantity for a null model, where individuals in each pair are randomly assigned to the urban or rural group, with the corresponding errors (standard deviations over 10000 realizations of the random model). **b)** Median relative difference innumber of unique apps across matched pairs of individuals, aggregated by age-group (see description of subplot b). **c)** Median relative difference in number of unique apps across matched pairs of individuals, aggregated by gender (see description of subplot b, errorbars are not visible due to their small size).

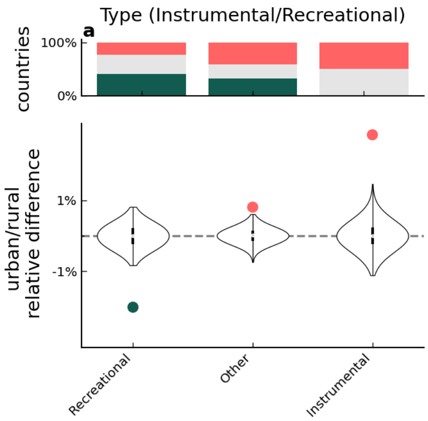

Fig. S16. Urban/rural differences in allocation of smartphone usage. Relative difference between urban and rural individuals in fraction of smartphone time by type of usage (recreational/instrumental). The bottom plot shows the distribution of the median difference in randomized pairs (violin plots), and the actual median difference (dots). Dots are colored in red when the difference is significantly positive (larger usage in urban individuals), in green when the difference is significantly negative (larger usage in rural individuals), and in gray elsewhere. The top plot shows the fraction of countries such that usage is significantly larger in urban (red bars) and rural (green bars) individuals. The fraction of countries with non-significant difference is displayed in gray.

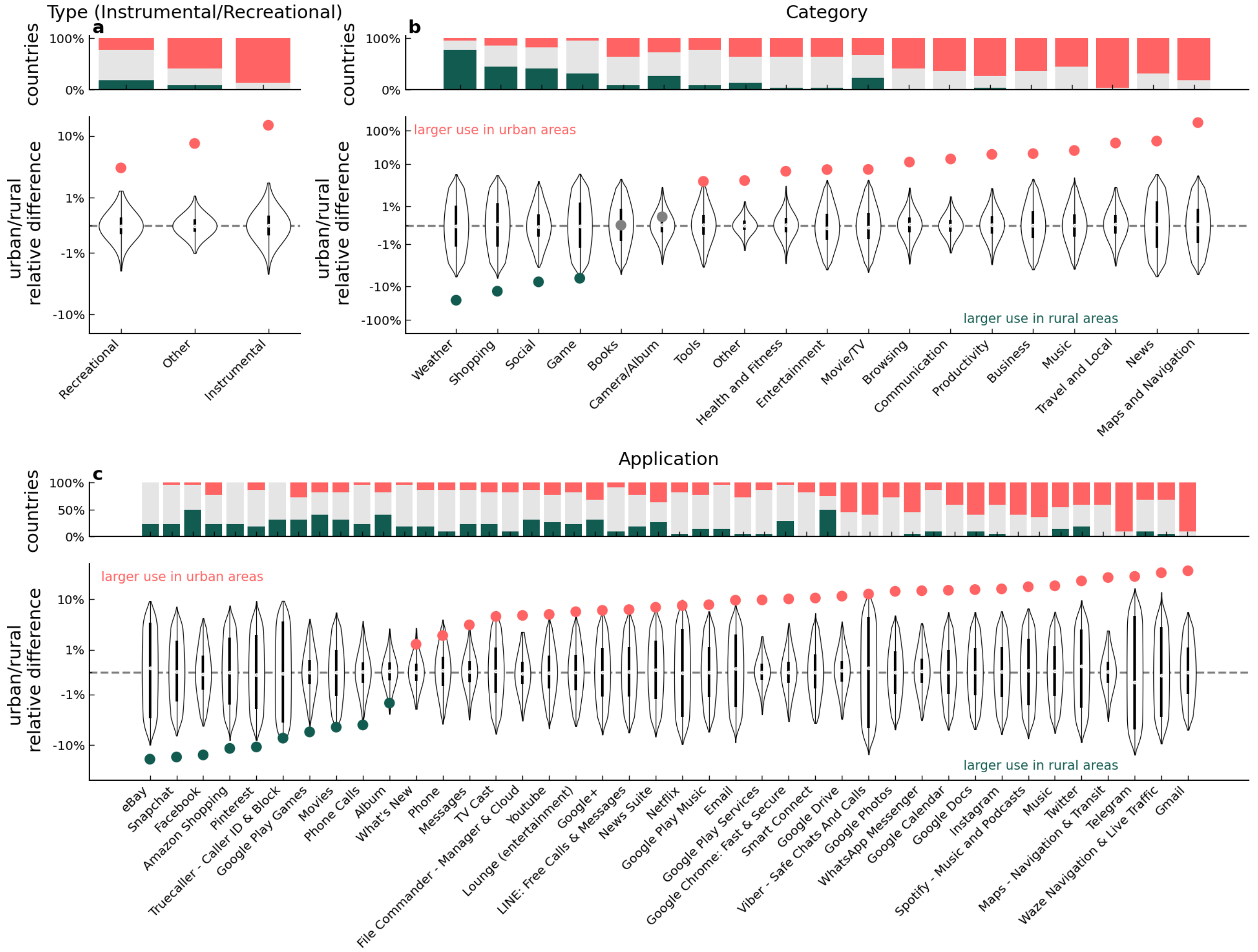

Fig. S17. Urban/rural differences in smartphone usage. Relative difference between urban and rural individuals in median daily smartphone time by type of usage (recreational/instrumental) **(a)**; category of application **(b)**; and single applications **(c)**. Here we show the typical usage in each category. The figure is described in the caption of Fig. 2.

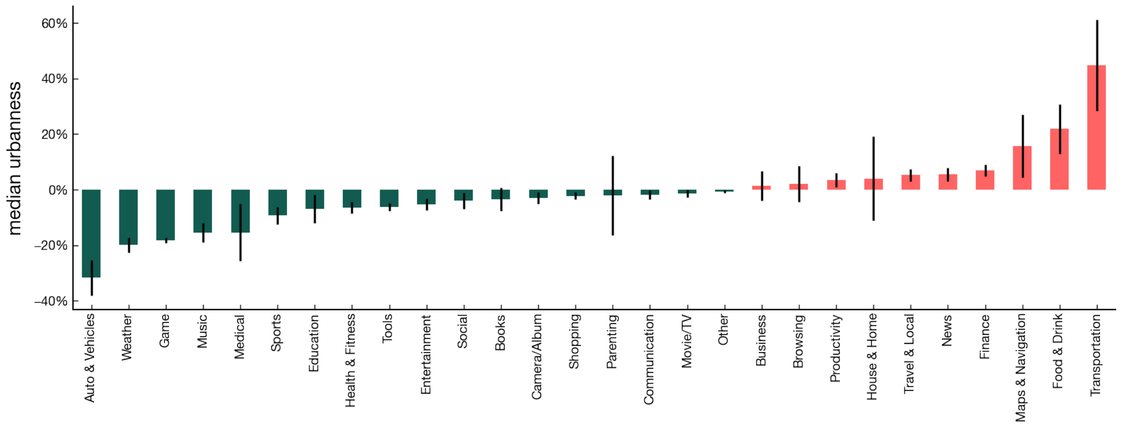

Fig. S18. Median *urbanness* by app category.

**Dep. Variable:**	median_screen_time	**R-squared:**	0.146
**Model:**	OLS	**Adj. R-squared:**	0.146
**Method:**	Least Squares	**F-statistic:**	9916.
**Date:**	Mon, 28 Aug 2023	**Prob (F-statistic):**	0.00
**Time:**	12:28:26	**Log-Likelihood:**	-2.7637e+06
**No. Observations:**	464455	**AIC:**	5.527e+06
**Df Residuals:**	464446	**BIC:**	5.527e+06
**Df Model:**	8		
**Covariance Type:**	nonrobust		

	**coef**	**std err**	**t**	**P> |t|**	**[0.025**	**0.975]**
**Intercept**	186.4165	0.458	407.264	0.000	185.519	187.314
**C(gender)[T.MALE]**	-15.5746	0.291	-53.562	0.000	-16.145	-15.005
**C(urbanization)[T.suburban]**	4.0772	0.515	7.920	0.000	3.068	5.086
**C(urbanization)[T.urban]**	7.1874	0.456	15.778	0.000	6.295	8.080
**age**	-32.7403	0.142	-230.581	0.000	-33.019	-32.462
**GID_0_0**	-7.8580	0.223	-35.191	0.000	-8.296	-7.420
**GID_0_1**	-1.6801	0.148	-11.326	0.000	-1.971	-1.389
**GID_0_2**	2.2021	0.146	15.078	0.000	1.916	2.488
**GID_0_3**	10.0843	0.208	48.497	0.000	9.677	10.492

**Omnibus:**	33140.071	**Durbin-Watson:**	2.003
**Prob(Omnibus):**	0.000	**Jarque-Bera (JB):**	40662.047
**Skew:**	0.707	**Prob(JB):**	0.00
**Kurtosis:**	3.318	**Cond. No.**	8.15

Table S3. OLS Model Results

Table S4. Mixed Linear Model Regression Results

Model:	MixedLM	Dependent Variable:	median_screen_time
No. Observations:	67175	Method:	REML
No. Groups:	22	Scale:	5489.9754
Min. group size:	670	Log-Likelihood:	-402378.4888
Max. group size:	24099	Converged:	Yes
Mean group size:	3053.4		

	Coef.	Std.Err.	z	P> |z|	[0.025	0.975]
Intercept	148.685	11.079	13.420	0.000	126.970	170.399
C(country_year)[T.AUS2016]	17.412	11.333	1.536	0.124	-4.800	39.624
C(country_year)[T.AUS2017]	26.551	11.154	2.380	0.017	4.690	48.412
C(country_year)[T.AUS2018]	26.175	12.243	2.138	0.033	2.178	50.171
C(country_year)[T.AUS2019]	41.123	15.589	2.638	0.008	10.568	71.677
C(country_year)[T.BRA2015]	35.535	11.611	3.060	0.002	12.778	58.292
C(country_year)[T.BRA2016]	50.134	11.377	4.407	0.000	27.835	72.432
C(country_year)[T.BRA2017]	59.408	11.289	5.262	0.000	37.281	81.535
C(country_year)[T.BRA2018]	51.638	11.535	4.476	0.000	29.029	74.247
C(country_year)[T.BRA2019]	45.678	13.317	3.430	0.001	19.577	71.779
C(country_year)[T.DEU2015]	-17.666	11.713	-1.508	0.131	-40.622	5.291
C(country_year)[T.DEU2016]	2.167	11.306	0.192	0.848	-19.992	24.326
C(country_year)[T.DEU2017]	13.004	11.221	1.159	0.246	-8.988	34.997
C(country_year)[T.DEU2018]	7.503	11.367	0.660	0.509	-14.777	29.782
C(country_year)[T.DEU2019]	16.032	11.979	1.338	0.181	-7.447	39.511
C(country_year)[T.EGY2015]	-6.992	19.650	-0.356	0.722	-45.506	31.522
C(country_year)[T.EGY2016]	44.268	13.452	3.291	0.001	17.903	70.634
C(country_year)[T.EGY2017]	51.928	12.647	4.106	0.000	27.140	76.715
C(country_year)[T.EGY2018]	46.209	13.383	3.453	0.001	19.979	72.439
C(country_year)[T.EGY2019]	60.715	17.069	3.557	0.000	27.260	94.171
C(country_year)[T.ESP2015]	7.165	12.636	0.567	0.571	-17.600	31.931
C(country_year)[T.ESP2016]	12.846	11.754	1.093	0.274	-10.191	35.883
C(country_year)[T.ESP2017]	24.207	11.592	2.088	0.037	1.487	46.926
C(country_year)[T.ESP2018]	22.778	11.901	1.914	0.056	-0.548	46.104
C(country_year)[T.ESP2019]	28.684	13.445	2.133	0.033	2.332	55.036
C(country_year)[T.FRA2015]	13.736	12.062	1.139	0.255	-9.906	37.378
C(country_year)[T.FRA2016]	11.675	11.448	1.020	0.308	-10.762	34.112
C(country_year)[T.FRA2017]	21.986	11.314	1.943	0.052	-0.189	44.162
C(country_year)[T.FRA2018]	16.361	11.497	1.423	0.155	-6.173	38.895
C(country_year)[T.FRA2019]	20.823	12.315	1.691	0.091	-3.314	44.960
C(country_year)[T.GBR2015]	14.783	11.758	1.257	0.209	-8.261	37.827
C(country_year)[T.GBR2016]	23.416	11.332	2.066	0.039	1.205	45.627
C(country_year)[T.GBR2017]	32.915	11.227	2.932	0.003	10.911	54.919
C(country_year)[T.GBR2018]	32.401	11.377	2.848	0.004	10.101	54.700
C(country_year)[T.GBR2019]	42.201	12.109	3.485	0.000	18.469	65.933
C(country_year)[T.IND2015]	54.830	13.567	4.041	0.000	28.238	81.421
C(country_year)[T.IND2016]	49.884	12.291	4.059	0.000	25.794	73.974
C(country_year)[T.IND2017]	45.337	11.812	3.838	0.000	22.187	68.488
C(country_year)[T.IND2018]	36.796	12.261	3.001	0.003	12.764	60.828
C(country_year)[T.IND2019]	22.094	14.832	1.490	0.136	-6.977	51.164
C(country_year)[T.IRN2015]	40.975	16.216	2.527	0.012	9.192	72.758
C(country_year)[T.IRN2016]	54.492	13.319	4.091	0.000	28.388	80.596
C(country_year)[T.IRN2017]	70.945	12.383	5.729	0.000	46.675	95.216
C(country_year)[T.IRN2018]	67.258	12.693	5.299	0.000	42.380	92.137
C(country_year)[T.IRN2019]	65.755	14.237	4.618	0.000	37.850	93.659
C(country_year)[T.ITA2015]	0.417	14.425	0.029	0.977	-27.856	28.691
C(country_year)[T.ITA2016]	15.690	12.842	1.222	0.222	-9.480	40.860
C(country_year)[T.ITA2017]	24.662	12.397	1.989	0.047	0.364	48.960
C(country_year)[T.ITA2018]	14.159	12.823	1.104	0.270	-10.973	39.291
C(country_year)[T.ITA2019]	16.584	15.196	1.091	0.275	-13.199	46.367
C(country_year)[T.JPN2015]	39.522	11.086	3.565	0.000	17.794	61.250
C(country_year)[T.JPN2016]	41.237	11.026	3.740	0.000	19.626	62.849
C(country_year)[T.JPN2017]	47.756	11.015	4.336	0.000	26.167	69.344
C(country_year)[T.JPN2018]	46.321	11.028	4.200	0.000	24.707	67.935
C(country_year)[T.JPN2019]	50.929	11.091	4.592	0.000	29.191	72.667
C(country_year)[T.KOR2015]	48.488	13.944	3.477	0.001	21.157	75.818
C(country_year)[T.KOR2016]	66.274	12.998	5.099	0.000	40.799	91.750
C(country_year)[T.KOR2017]	67.962	12.345	5.505	0.000	43.765	92.158
C(country_year)[T.KOR2018]	65.337	12.798	5.105	0.000	40.253	90.421
C(country_year)[T.KOR2019]	77.274	15.256	5.065	0.000	47.372	107.176

C(country_year)[T.MEX2015]	36.193	13.011	2.782	0.005	10.692	61.693
C(country_year)[T.MEX2016]	62.220	12.065	5.157	0.000	38.573	85.868
C(country_year)[T.MEX2017]	69.610	11.797	5.901	0.000	46.489	92.731
C(country_year)[T.MEX2018]	65.764	12.189	5.395	0.000	41.874	89.653
C(country_year)[T.MEX2019]	55.502	14.052	3.950	0.000	27.960	83.044
C(country_year)[T.NLD2015]	0.039	14.452	0.003	0.998	-28.287	28.364
C(country_year)[T.NLD2016]	15.205	12.384	1.228	0.220	-9.067	39.477
C(country_year)[T.NLD2017]	21.707	12.029	1.805	0.071	-1.869	45.282
C(country_year)[T.NLD2018]	18.816	12.504	1.505	0.132	-5.691	43.324
C(country_year)[T.NLD2019]	22.203	14.158	1.568	0.117	-5.547	49.952
C(country_year)[T.NOR2015]	17.129	14.674	1.167	0.243	-11.631	45.889
C(country_year)[T.NOR2016]	13.957	13.023	1.072	0.284	-11.568	39.482
C(country_year)[T.NOR2017]	22.668	12.601	1.799	0.072	-2.029	47.366
C(country_year)[T.NOR2018]	28.615	13.514	2.117	0.034	2.128	55.102
C(country_year)[T.NOR2019]	49.238	16.742	2.941	0.003	16.425	82.052
C(country_year)[T.POL2015]	-12.892	13.614	-0.947	0.344	-39.575	13.791
C(country_year)[T.POL2016]	4.131	12.260	0.337	0.736	-19.898	28.161
C(country_year)[T.POL2017]	12.355	11.887	1.039	0.299	-10.943	35.653
C(country_year)[T.POL2018]	2.163	12.255	0.177	0.860	-21.856	26.182
C(country_year)[T.POL2019]	0.940	14.172	0.066	0.947	-26.836	28.716
C(country_year)[T.RUS2015]	8.548	12.015	0.711	0.477	-15.001	32.098
C(country_year)[T.RUS2016]	33.129	11.483	2.885	0.004	10.623	55.636
C(country_year)[T.RUS2017]	33.888	11.368	2.981	0.003	11.608	56.169
C(country_year)[T.RUS2018]	24.687	11.563	2.135	0.033	2.024	47.351
C(country_year)[T.RUS2019]	32.791	12.525	2.618	0.009	8.242	57.340
C(country_year)[T.SWE2015]	2.413	12.607	0.191	0.848	-22.297	27.122
C(country_year)[T.SWE2016]	15.871	11.820	1.343	0.179	-7.296	39.038
C(country_year)[T.SWE2017]	22.831	11.672	1.956	0.050	-0.046	45.709
C(country_year)[T.SWE2018]	20.902	11.954	1.749	0.080	-2.527	44.331
C(country_year)[T.SWE2019]	22.935	12.985	1.766	0.077	-2.515	48.385
C(country_year)[T.TUR2015]	22.681	12.671	1.790	0.073	-2.154	47.516
C(country_year)[T.TUR2016]	56.250	11.722	4.799	0.000	33.274	79.225
C(country_year)[T.TUR2017]	58.824	11.414	5.154	0.000	36.454	81.194
C(country_year)[T.TUR2018]	52.919	11.583	4.569	0.000	30.218	75.621
C(country_year)[T.TUR2019]	60.777	12.356	4.919	0.000	36.559	84.994
C(country_year)[T.TWN2015]	65.813	11.972	5.497	0.000	42.349	89.277
C(country_year)[T.TWN2016]	77.424	11.496	6.735	0.000	54.892	99.957
C(country_year)[T.TWN2017]	79.344	11.387	6.968	0.000	57.025	101.663
C(country_year)[T.TWN2018]	73.182	11.558	6.332	0.000	50.530	95.834
C(country_year)[T.TWN2019]	68.872	12.251	5.622	0.000	44.861	92.883
C(country_year)[T.USA2015]	35.218	14.081	2.501	0.012	7.620	62.817
C(country_year)[T.USA2016]	36.184	12.884	2.809	0.005	10.933	61.436
C(country_year)[T.USA2017]	53.124	12.093	4.393	0.000	29.421	76.827
C(country_year)[T.USA2018]	48.134	12.545	3.837	0.000	23.546	72.721
C(country_year)[T.USA2019]	73.185	15.794	4.634	0.000	42.230	104.140
C(country_year)[T.ZAF2015]	8.973	16.052	0.559	0.576	-22.489	40.435
C(country_year)[T.ZAF2016]	23.470	13.675	1.716	0.086	-3.333	50.272
C(country_year)[T.ZAF2017]	32.730	12.779	2.561	0.010	7.682	57.777
C(country_year)[T.ZAF2018]	19.211	13.525	1.420	0.155	-7.297	45.719
C(country_year)[T.ZAF2019]	42.285	17.576	2.406	0.016	7.837	76.732
C(gender)[T.MALE]	-15.745	1.208	-13.036	0.000	-18.113	-13.378
C(urbanization)[T.2]	0.113	2.155	0.053	0.958	-4.111	4.338
C(urbanization)[T.3]	4.757	1.933	2.461	0.014	0.968	8.546
age	-30.069	0.601	-50.060	0.000	-31.246	-28.892
useruuid Var	6407.330	1.304				

References

1. Alexis Hiniker, Shwetak N Patel, Tadayoshi Kohno, and Julie A Kientz. Why would you do that? predicting the uses and gratifications behind smartphone-usage behaviors. In *Proceedings of the 2016 ACM International Joint Conference on Pervasive and Ubiquitous Computing*, pages 634–645, 2016.
2. Elizabeth A Stuart. Matching methods for causal inference: A review and a look forward. *Statistical science: a review journal of the Institute of Mathematical Statistics*, 25(1):1, 2010.
3. Cheng Guo and Felix Berkhahn. Entity embeddings of categorical variables. *arXiv preprint arXiv:1604.06737*, 2016.
